# Comparison of the health-related outcomes for traditional cigarettes, e-cigarettes, heat-not-burn cigarettes and snus: a systematic review and meta-analysis

**DOI:** 10.1186/s12889-026-27067-z

**Published:** 2026-03-26

**Authors:** Paulina Natalia Kopa-Stojak, Marharyta Sobczak, Rafal Pawliczak

**Affiliations:** Department of Immunopathology, Faculty of Medicine, Medical University of Lodz, Lodz, Poland

**Keywords:** Health, Smoking, Vaping, Smokeless tobacco, Pulmonary disease, Cardiovascular disease, Metabolic disease

## Abstract

**Background:**

Tobacco products using is a global public health problem. The aim of this systematic review and meta-analysis was to assessed the health-related outcomes associated with acute and chronic using of traditional cigarettes (TCs), e-cigarettes (ECs), heated tobacco products (HTPs) and snus.

**Methods:**

PubMed, EMBASE, Web of Science, Scopus and the Cochrane Central Register of Controlled Trials databases were searched by the terms: ‘cigarette’, ‘conventional cigarette’, ‘ENDS’, ‘electronic nicotine delivery system’, ‘electronic cigarette’, ‘e-cigarette’, ‘heat-not-burn product’, ‘heat-not-burn cigarette’, ‘tobacco heating product’, ‘heated tobacco product’, ‘snus’, ‘Swedish snuff’, ‘oral nicotine pouches’, ‘nicotine pouches’, ‘health’, ‘health effect’, ‘health impact’. Random effects model was used to calculate effect sizes. I^2^ statistic was used to evaluate the heterogeneity of studies. Funnel plot and Egger’s regression test were used to assess publication bias. Statistical analysis of the data was performed in R (version 4.2.2).

**Results:**

Acute exposure to analyzed tobacco products significantly affects heart rate (MD = 6.78; 95% CI [4.86; 8.69]; *p* < 0.0001; I^2^ = 49%). Chronic exposure to such tobacco products significantly affects triglycerides level (MD = 18.09; 95% CI [6.12; 30.07]; *p* = 0.003; I^2^ = 62%), systolic blood pressure (MD = 6.99; 95% CI [1.62; 12.37]; *p* = 0.01; I^2^ = 78%) and diastolic blood pressure (MD = 5.2; 95% CI [2.27; 8.13]; *p* = 0.0005; I^2^ = 54%). There was a greater odds of cancer with chronic smoking by 32% (OR = 1.32; 95% CI [1.08; 1.61]; *p* = 0.009; I^2^ = 92%). We have shown that former smokers have a 5% higher chance of developing cancer, while current smokers have an increased chance by as much as 61%.

**Conclusions:**

Robust evidence supports adverse health effects of traditional cigarettes, whereas evidence for alternative products remains limited and heterogeneous. Therefore, longitudinal studies involving individuals exclusively using conventional and alternative tobacco products should be performed in the future.

**Trial registration:**

The study protocol of this systematic review was registered in International Prospective Register of Systematic Reviews (PROSPERO) with registration number CRD420251062406.

**Supplementary Information:**

The online version contains supplementary material available at 10.1186/s12889-026-27067-z.

## Background

Tobacco products using is a global public health problem. According to the World Health Organization (WHO) statistics, there is over 1.25 billion of smokers (almost 21% of people ≥ 15 years-old) worldwide. Moreover, tobacco use is responsible for over 8 million of deaths (including 1.3 million associated with second-hand exposure to cigarette smoke) annually [1, 2]. There is at least 7,000 chemical constituents of cigarette smoke, including: tobacco-specific nitrosamines (TSNAs), polycyclic aromatic hydrocarbons (PAHs), volatile organic compounds (VOCs), heavy metals, tar, carbon monoxide (CO) and huge amount of free radicals (up to 10^15^-10^17^ particles/puff) [3–5]. Furthermore, at least 93 of cigarette smoke components are classified as a carcinogen for humans (IARC group 1), such as benzene, and probably or possibly carcinogenic for humans (IARC group 2 A and 2B), such as benzo[a]pyrene, formaldehyde, acetaldehyde or 4-(methylnitrosamino)-1-(3-pyridyl)-1-butanone (NNK) [6–8]. Toxic components of cigarette smoke directly impact structural damage by peroxidation of proteins, DNA and lipids. Moreover, cigarette smoke components leading to activation of structural cells, infiltration of pro-inflammatory cells [9, 10], increase in release of cytokines, chemokines and up-regulation of pro-inflammatory and adhesion molecules [11].

In addition to classic tobacco products (i.e. cigarettes, cigars, water pipes, etc.), tobacco market offers a wide range of alternative products, including electronic nicotine delivery system (ENDS, also known as electronic cigarettes (ECs)), heated tobacco products (HTPs) and smokeless tobacco products (e.g. snus, moist snuff, chewing tobacco). Such alternative products offered reduction in the exposure to hazardous and potentially hazardous constituents (HPHCs) presented in the smoke from traditional cigarettes (TCs), due to limiting chemical components of such products, as well as avoiding combustion of tobacco [12–14]. Despite a significant restrictions on hazardous substances, alternative tobacco products may contain similar or higher nicotine concentrations compared to traditional cigarettes (up to 20 mg/mL or 59 mg/mL for ECs on EU and US market, respectively; 1.1–1.2 mg/stick for HTP; 6.8–20.6 mg/g for snus) [15–17]. Furthermore, such alternative tobacco products are becoming increasingly popular among young people and never-smokers. In this condition their usage may be unsafe and may increase probability of developing tobacco-related diseases or nicotine addiction [18–25].

Current literature confirms the negative health effects associated with smoking. Tobacco products using increased risk of many disorders, including pulmonary diseases (i.e. chronic obstructive pulmonary disease (COPD), idiopathic pulmonary fibrosis (IPF), asthma exacerbation), cardiovascular diseases (i.e. coronary heart disease, stroke, hypertension, atherosclerosis), neurological disorders (i.e. Alzheimer’s disease, dementia), metabolic disorders (i.e. diabetes mellitus), reproductive disorders (i.e. infertility), chronic inflammatory disorders (i.e. rheumatoid arthritis (RA)) [26–28] and cancers (i.e. lung, larynx, mouth, esophagus, throat, bladder, kidney, liver, stomach, pancreas, colon, cervix cancer or acute myeloid leukemia) [29, 30]. Moreover, smoking affects bones, teeth and gums, reduces fertility and increases risk of many birth defects [31] as well as leading to nicotine dependence [32]. The aim of this systematic review and meta-analysis was to assessed the health-related outcomes associated with acute and chronic using of traditional cigarettes, e-cigarettes, heated tobacco products and snus.

## Methods

### Eligibility criteria

The study protocol of this systematic review and meta-analysis was carried out in accordance with Preferred Reporting Items for Systematic Reviews and Meta-Analyses (PRISMA) guidelines [33] and registered in the International Prospective Register of Systematic Reviews (PROSPERO) with registration number CRD420251062406. This systematic review and meta-analysis analyzes the health-related outcomes associated with smoking of conventional cigarettes, e-cigarettes, heat-not-burn cigarettes and using of snus. Selection of the publications for this systematic review and meta-analysis was based on pre-determined inclusion and exclusion criteria. The inclusion criteria were: study type (randomized and non-randomized controlled, case-control, cross-sectional), age of participants (adolescents (12–17 years-old), young adults (18–24 years-old) and adults (> 24 years-old)), tobacco product type (TC, EC, HTP, snus), exposure (acute (refers to a short-term exposure (one-off or up to few days), often resulting in immediate effects)), chronic (refers to long-term, repeated exposure (weeks/months/years) that can lead to gradual health issues over time)), measuring outcomes (health-related outcomes: heart rate (HRT), systolic blood pressure (SBP), diastolic blood pressure (DBP), forced expiratory volume in one second (FEV_1_), forced vital capacity (FVC), total cholesterol (CHOL), triglycerides (TRI) and glucose (GLU) level, odds ratio (OR) of association between smoking and cancers) and study language (English).

### Search strategy

PubMed, EMBASE, Web of Science, Scopus and the Cochrane Central Register of Controlled Trials databases were searched to find literature published up to July 15, 2025. The following keywords were used: cigarette, conventional cigarette, ENDS, electronic nicotine delivery system, electronic cigarette, e-cigarette, heat-not-burn product, heat-not-burn cigarette, tobacco heating product, heated tobacco product, snus, Swedish snuff, oral nicotine pouches, nicotine pouches, health, health effect, health impact. Full search strategy for each database is presented in *Additional file 1*.

### Study selection

This systematic review and meta-analysis focuses on human studies that analyzed health-related outcomes associated with acute and chronic use of conventional cigarettes, e-cigarettes, heat-not-burn cigarettes and snus. Two authors (P.N. K-S. and M.S.) conducted an independent selection of publications by the title and abstract, to select potentially relevant human studies to be further analyzed. The exclusion criteria were: publication type (letter to the editor, commentary, review/systematic review, case series, surveys, protocols), language (other than English), tobacco product type (other than TC, EC, HTP or snus, i.e. waterpipe, cigars) and aims of publication (opinions/subjective assessments of harmfulness of tobacco products, chemical composition, analytical methods, market size analysis, regulatory frameworks, prevalence of use). In addition, the studies which analyzed tobacco products in general (or group of products), without division into specific types (TC, EC, HTP, snus), studies with poly-users of tobacco products, studies which investigated biomarkers of exposure (BoE) or biomarkers of potential hazard (BoPH) or other unrelated studies were excluded from further analysis. The consensus in the case of any discrepancies between the authors’ opinions on relevance of some individual studies and their inclusion into further analysis was achieved by discussion.

### Data extraction and synthesis

The key data extracted from each relevant study were: author(s) and the year of publication, study design, study location, sample size, age and sex of the participants, exposure type and health-related outcomes: heart rate (HRT), systolic blood pressure (SBP), diastolic blood pressure (DBP), forced expiratory volume in one second (FEV_1_), forced vital capacity (FVC), total cholesterol (CHOL) level (mg/dl), triglycerides (TRI) level (mg/dl), glucose (GLU) level (mg/dl) and odds ratio (OR) of association between smoking and cancers. Continuous data was converted into mean and standard deviation [mean (SD)], if it was presented as mean and standard error.

### Quality assessment

The quality of randomized clinical trials was assessed using the Cochrane Collaboration’s tool for assessing risk of bias in randomized trials [34]. The following criteria were used: random sequence generation, allocation concealment, blinding of participants and personnel, blinding of outcome assessment, incomplete outcome data, selective reporting and other bias (assessed at 3 levels such as low, high or unclear risk). For assessing the quality of nonrandomized studies, we used the Newcastle-Ottawa Scale (NOS) [35], while to assess the quality of cross-sectional studies we used the Appraisal tool for Cross-Sectional Studies (AXIS) tool [36].

### Statistical analysis

Statistical analysis of the data was performed in R (version 4.2.2). To compare the effect of different types of smoking in the experimental group compared to the control group, the mean difference (MD) with 95% confidence intervals (95% CI) was calculated for continuous outcomes. An OR with 95% CI was also used as a measure of the cumulative effect. Random effects model was used to calculate effect sizes. I^2^ statistic was used to evaluate the heterogeneity of studies: I^2^ < 40% may not be important; 30% < I^2^ < 60% means moderate heterogeneity; 50% < I^2^ < 90% means substantial heterogeneity; I^2^ > 75% means considerable heterogeneity [37]. Funnel plot and Egger’s regression test were used to assess publication bias. The results of this meta-analysis were considered statistically significant at *p* < 0.05.

## Results

### Study selection

**Fig. 1:**
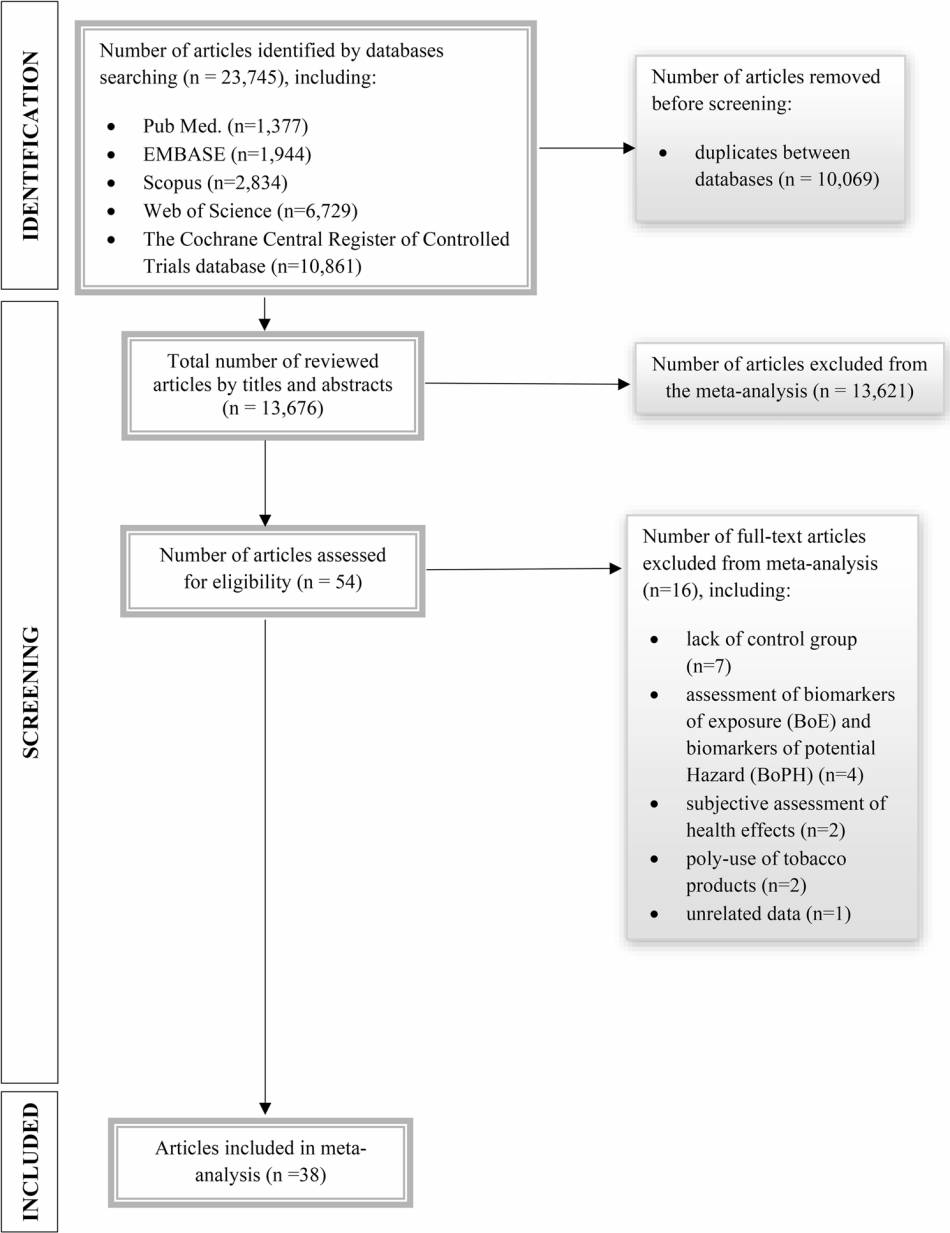
Article selection process recommended by preferred reporting items for Systematic Reviews and Meta-Analyses (PRISMA) statement

The literature search allowed to identify *n* = 23,745 potential articles (including *n* = 1,377 in Pub Med, *n* = 1,944 in EMBASE, *n* = 2,834 in Scopus, *n* = 6,729 in Web of Science and *n* = 10,861 in the Cochrane Central Register of Controlled Trials database). Removing duplicate publications between the searched databases allowed to reduce the number of publications to *n* = 13,676, which were analyzed by the title and abstract. Then, the number of articles was reduced to 54, which were assessed for eligibility. Finally, after full-text screening, 38 articles were qualified for this systematic review and meta-analysis. Searching strategy of article identification, as recommended by Preferred Reporting Items for Systematic Reviews and Meta-Analyses (PRISMA) Statement is presented in Fig. 1.

### Studies characteristics

Studies characteristics are presented in Table 1. Among studies included to this meta-analysis, 14 was carried out in North America [38–51], 10 in Asia [52–61], 11 in Europe [62–72], 1 in Africa [73], 1 in Australia and Oceania [74] and 1 internationally (North America, Europe, Asia, Australia) [75]. Furthermore, 11 studies investigated cancer incidences among tobacco products users [43–49, 53, 62, 63, 75], 12 cardiovascular parameters [38, 39, 41, 52, 56, 61, 64, 66–70], 14 respiratory parameters [39, 51, 52, 54–60, 67, 72–74] and 6 metabolic parameters [40, 42, 50, 61, 65, 71]. In addition, 30 studies investigated health-related outcomes for TCs [39, 40, 42–50, 52, 53, 55–66, 71–75], 8 for ECs [38, 39, 41, 50, 51, 54, 67, 69], 4 for HTP [60, 66, 70, 73] and 1 for snus [68]. A summary detailing the number of studies and participants per product and per outcome are presented in *Additional file 2*.

**Table 1:** Characteristic of studies included to systematic review and meta-analysis

No.	Author(s), year of publication	Study design	Study location	Participants	Sample size	Tobacco product	Exposure type	Measured outcomes(s)
1	Gonzales and Cooke, 2021 [38]	a randomized crossover controlled trial	USA	healthy young adults (21 ± 1 years-old) (9 males, 6 females)	*n* = 15	e-cigarette (EC)	acute	heart rate (HRT); systolic blood pressure (SBP); diastolic blood pressure (DBP)
2	Lyytinen et al. 2023 [69]	a randomized, double-blind crossover trial	Sweden	healthy young adult and adult (18–45 years-old) occasional cigarette smokers (max 10 cigarettes per month) or snus users (max 10 pouches per month)(7 males, 15 females)	*n* = 22	e-cigarette (EC)	acute	heart rate (HRT); systolic blood pressure (SBP); diastolic blood pressure (DBP)
3	Antoniewicz et al. 2019 [67]	a randomized double-blinded crossover trial	Sweden	healthy adult (26 ± 3 years-old) occasional cigarette smokers (max 10 cigarettes per month)(6 males, 9 females)	*n* = 15	e-cigarette (EC)	acute	heart rate (HRT); systolic blood pressure (SBP); diastolic blood pressure (DBP); forced expiratory volume in 1 s (FEV1); forced vital capacity (FVC)
4	D’Ruiz et al. 2017 [39]	a randomized, open-label, forced-switch parallel arm study	USA	healthy adult (21–65 years old) smokers (≥ 10 cigarettes per day, ≥ 1 year) (68 males, 37 females)	*n* = 105	traditional cigarette (TC); e-cigarette (EC)	acute	heart rate (HRT); systolic blood pressure (SBP); diastolic blood pressure (DBP); forced expiratory volume in 1 s (FEV1); forced vital capacity (FVC)
5	Price et al. 2003 [40]	clinical trial with two-arms	USA	healthy adult (24 ± 2 years-old) smokers (≥ 20 cigarettes per day) and non-smokers (23 ± 1 years-old) (18 males)	*n* = 18	traditional cigarette (TC)	chronic	glucose level
6	Sobczak et al. 2014 [71]	a cross-sectional study	Poland	healthy young adult and adult (20–60 years-old) smokers and EC users (231 males)	*n* = 231	traditional cigarette (TC)	chronic	total cholesterol; triglycerides
7	Lyytinen et al. 2024 [70]	a randomized crossover study	Sweden	healthy young adults and adults (20–40 years-old) with occasional tobacco use (max 10 cigarettes per month or 10 snus pouches per month)(12 males, 11 females)	*n* = 23	heated tobacco product (HTP)	acute	heart rate (HRT); systolic blood pressure (SBP); diastolic blood pressure (DBP)
8	Boakye et al. 2023 [41]	a cross-sectional study	USA	healthy adults(24.3 ± 4.0 years-old) non-smokers (< 5 times EC use) and EC users (≥ 6 months)(36 males, 10 females)	*n* = 46	e-cigarette (EC)	chronic	heart rate (HRT); systolic blood pressure (SBP); diastolic blood pressure (DBP)
9	Wu et al. 2020 [42]	a cross-sectional study	USA and Canada	older cigarette smokers (74.7 ± 5.9 years-old) and non-smokers (75 ± 5.3 years-old)(209 males, 206 females)	*n* = 415	traditional cigarette (TC)	chronic	total cholesterol; triglycerides; glucose level
10	Antoniewicz et al. 2018 [68]	a randomized double-blind crossover study	Sweden	healthy young adult and adult (≥ 18 years-old) snus users (12 males, 17 females)	*n* = 29	snus	acute	heart rate (HRT); systolic blood pressure (SBP); diastolic blood pressure (DBP)
11	Unverdorben et al. 2010 [73]	a randomized three-period cross-over	South Africa	healthy adult (45.1 ± 7.1 years-old) smokers (20–40 cigarettes per day, ≥ 10 years)	*n* = 57	traditional cigarette (TC); heated tobacco product (HTP)	acute	forced expiratory volume in 1 s (FEV1); forced vital capacity (FVC)
12	Ikonomidis et al. 2021 [66]	a randomized crossover study	Greece	healthy young adult and adult (≥ 18 years-old) smokers (38 ± 18 packs per year)(34 males, 41 females)	*n* = 75	traditional cigarette (TC); heated tobacco product (HTP)	acute	heart rate (HRT); systolic blood pressure (SBP); diastolic blood pressure (DBP)
13	Batic-Mujanovic et al. 2008 [65]	a prospective, randomized controlled study	Bosnia	healthy adult (30–70 years-old) smokers (9.75 ± 3.8 years) and non-smokers (45 males, 60 females)	*n* = 105	traditional cigarette (TC)	chronic	total cholesterol; triglycerides
14	Lanza et al. 2015 [64]	a comparative study	Italy	healthy young adult and adult (≥ 18 years-old) smokers (min. 10 cigarettes per day) and non-smokers (12 males, 12 females)	*n* = 24	traditional cigarette (TC)	chronic	heart rate (HRT); systolic blood pressure (SBP); diastolic blood pressure (DBP)
15	Chen et al. 2015 [52]	a randomized crossover study	Taiwan	healthy young adult (20.4 ± 1.5 years-old) smokers (4.0 ± 2 years)(14 males)	*n* = 14	traditional cigarette (TC)	acute	heart rate (HRT); forced expiratory volume in 1 s (FEV1); forced vital capacity (FVC)
16	Meo et al. 2019 [54]	a cross-sectional study	Saudi Arabia	young healthy male young adult and adult (≥ 18 years-old) EC smokers and non-smokers (60 males)	*n* = 60	e-cigarette (EC)	chronic	forced expiratory volume in 1 s (FEV1); forced vital capacity (FVC)
17	Walter et al. 1979 [55]	a case-control study	India	healthy young adult (≥ 18 years-old) smokers and non-smokers (102 males)	*n* = 102	traditional cigarette (TC)	chronic	forced expiratory volume in 1 s (FEV1); forced vital capacity (FVC)
18	Habib et al. 2011 [56]	a case-control study	Saudi Arabia	healthy young adult (≥ 20 years-old) smokers and non-smokers (48 males)	*n* = 48	traditional cigarette (TC)	chronic	systolic blood pressure (SBP); diastolic blood pressure (DBP); forced expiratory volume in 1 s (FEV1); forced vital capacity (FVC)
19	Milaat and el-Ganai 1998 [57]	a cross-sectional study	Saudi Arabia	healthy young adult and adult (≥ 18 years-old) smokers and non-smokers (676 males)	*n* = 676	traditional cigarette (TC)	chronic	forced expiratory volume in 1 s (FEV1); forced vital capacity (FVC)
20	Jawed et al. 2012 [58]	a cross-sectional study	Pakistan	healthy young adult and adult (19–25 years-old) smokers (ex-smokers, current light smokers who smoked 1–20 cigarettes per day and current heavy smokers who smoked > 20 cigarettes per day) and non-smokers (244 males)	*n* = 244	traditional cigarette (TC)	chronic	forced expiratory volume in 1 s (FEV1); forced vital capacity (FVC)
21	Barter et al. 1985 [72]	a case-control study	UK	healthy middle-aged (44–61 years-old) smokers (24.1 cigarettes per day) and non-smokers (89 males)	*n* = 89	traditional cigarette (TC)	chronic	forced expiratory volume in 1 s (FEV1)
22	Byerley et al. 1992 [74]	a case-control study	Solmon Island	healthy adult (25–75 years-old) smokers and non-smokers (193 males, 201 females)	*n* = 394	traditional cigarette (TC)	chronic	forced expiratory volume in 1 s (FEV1); forced vital capacity (FVC)
23	Tantisuwat and Thaveeratitham 2014 [59]	a case-control study	Thailand	healthy young adult (15–18 years-old) smokers (1–3 years) and non-smokers (68 males)	*n* = 68	traditional cigarette (TC)	chronic	forced expiratory volume in 1 s (FEV1); forced vital capacity (FVC)
24	Harada et al. 2021 [60]	a prospective population-based study	Japan	health middle-aged (> 50 years-old)smokers (current, ex-smokers), HTTP users and dual users (resident population) (1,177 males, 1,435 females)	*n* = 2612	traditional cigarette (TC); heated tobacco product (HTP)	chronic	forced expiratory volume in 1 s (FEV1)
25	Kizhakke Puliyakote et al. 2021 [51]	a cross-sectional study	USA	healthy young adult and adult (≥ 20 years-old) EC users (> 1 year) and non-users (6 males, 3 females)	*n* = 9	e-cigarette (EC)	chronic	forced expiratory volume in 1 s (FEV1); forced vital capacity (FVC)
26	Chandala and Reddy 2014 [61]	a cross-sectional study	India	healthy and hypertensive middle-aged (45–50 years-old) smokers (11–20 cigarettes per day, 10 years) and non-smokers (150 males)	*n* = 150	traditional cigarette (TC)	chronic	heart rate (HRT); systolic blood pressure (SBP); diastolic blood pressure (DBP); total cholesterol; triglycerides; glucose level
27	Majid et al. 2021 [50]	a cross-sectional study	USA	healthy young adult and adult (21–45 years-old) smokers ≥ 100 cigarettes smoked in life), EC smokers (> 3 months), dual users and non-smokers (290 males, 235 females)	*n* = 525	e-cigarette (EC); EC + TC (dual use)	chronic	total cholesterol; triglycerides; glucose level
28	Kessides et al. 2011 [43]	a population-based case-control study	USA	healthy (164 patients) and cancer (82 patients) adult (40–85 years-old) smokers and never-smokers (126 males, 120 females)	*n* = 246	traditional cigarette (TC)	chronic	OR with 95% CI for melanoma
29	Jiang et al. 2012 [44]	a population-based case-control study	USA	healthy (1,586 patients) and cancer (1,439 patients) adult (25–64 years-old) smokers and never-smokers (2,353 males, 672 females)	*n* = 3,025	traditional cigarette (TC)	chronic	OR with 95% CI for bladder cancer
30	Bosetti et al. 2012 [75]	12 case-control studies within the International Pancreatic Cancer Case-Control Consortium (PanC4)	North America (USA, Canada), Europe (Italy), China, Australia	healthy (12,890 patients) and cancer (6,507 patients) adult (28–97 years-old) smokers and never-smokers (3,643 males, 15,754 females)	*n* = 19,397	traditional cigarette (TC)	chronic	OR with 95% CI for pancreatic cancer
31	Modugno et al. 2002 [45]	a population-based case-control study	USA	healthy (1,367 patients) and cancer (767 patients) young adult and adult (20–69 years-old) smokers and never-smokers (2,134 females)	*n* = 2,134	traditional cigarette (TC)	chronic	OR with 95% CI for ovarian cancer
32	Ellis et al. 2024 [46]	a cross-sectional study	USA	healthy (979 patients) and cancer (1,279 patients) adult (40–79 years-old) smokers and never-smokers (2,258 males)	*n* = 2,258	traditional cigarette (TC)	chronic	OR with 95% CI for prostate cancer
33	Connor et al. 2016 [47]	a consortium of three population-based case-control studies	USA	healthy (4,137 patients) and cancer (3,780 patients) adult (25–70 years-old) smokers and never-smokers (7,917 females)	*n* = 7,917	traditional cigarette (TC)	chronic	OR with 95% CI for breast cancer
34	Morton et al. 2003 [48]	a population-based case-control study	USA	healthy (718 patients) and cancer (601 patients) young adult and adult (21–84 years-old) smokers and never-smokers (1,319 females)	*n* = 1,319	traditional cigarette (TC)	chronic	OR with 95% CI for non-Hodgkin lymphoma (NHL)
35	Hjalgrim et al. 2007 [63]	a population-based case-control study	Denmark, Sweden	healthy (3,187 patients) and cancer (3,773 patients (3,055 NHL, 586 HL)) young adult and adult (18–74 years-old) smokers and never-smokers (2,164 males, 4,796 females)	*n* = 6960	traditional cigarette (TC)	chronic	OR with 95% CI for non-Hodgkin lymphoma (NHL); OR with 95% CI for Hodkin lymphoma (HL)
36	Chang et al. 2017 [53]	a population-based case-control study	China	healthy (2,595 patients) and cancer (2,530 patients) young adult and adult (20–74 years-old) smokers and never-smokers (3,764 males, 1,361 females)	*n* = 5,125	traditional cigarette (TC)	chronic	OR with 95% CI for nasopharyngeal carcinoma (NPC)
37	Yang et al. 2010 [62]	a population-based case-control study	Poland	healthy (1,925 patients) and cancer (551 patients) young adult and adult (20–74 years-old) smokers and never-smokers (2,476 females)	*n* = 2,476	traditional cigarette (TC)	chronic	OR with 95% CI for endometrial cancer
38	Boffetta et al. 2011 [49]	seven case-control studies	USA	healthy (12,012 patients) and cancer (799 patients) adult (45–73 years-old) smokers and never-smokers (6,518 males, 6,293 females)	*n* = 12,811	traditional cigarette (TC)	chronic	OR with 95% CI for bronchioloalveolar carcinoma (BAC)

*Abbreviations: BAC* bronchioloalveolar carcinoma, *CI* confidence interval, *DBP* diastolic blood pressure, *EC* electronic cigarette, *FEV1* forced expiratory volume in one second, *FVC* forced vital capacity, *HL* Hodgkin lymphoma, *HTP* heated tobacco product, *HRT* heart rate, *NHL* non-Hodgkin lymphoma, *NPC* nasopharyngeal cancer, *OR *odds ratio, *SBP *systolic blood pressure, *TC* traditional cigarette

### Quality assessment

Among 9 included RCTs, 5 of studies have high risk of bias, while 4 indicated low risk, as shown on *Additional file 3*. *Additional file 4* shows the quality assessment of the included case-control studies using the Newcastle-Ottawa Scale, while *Additional file 5* shows the quality assessment of the included cross-sectional studies using the AXIS tool.

### The effect of acute smoking on cardiovascular parameters and pulmonary function

**Fig. 2:**
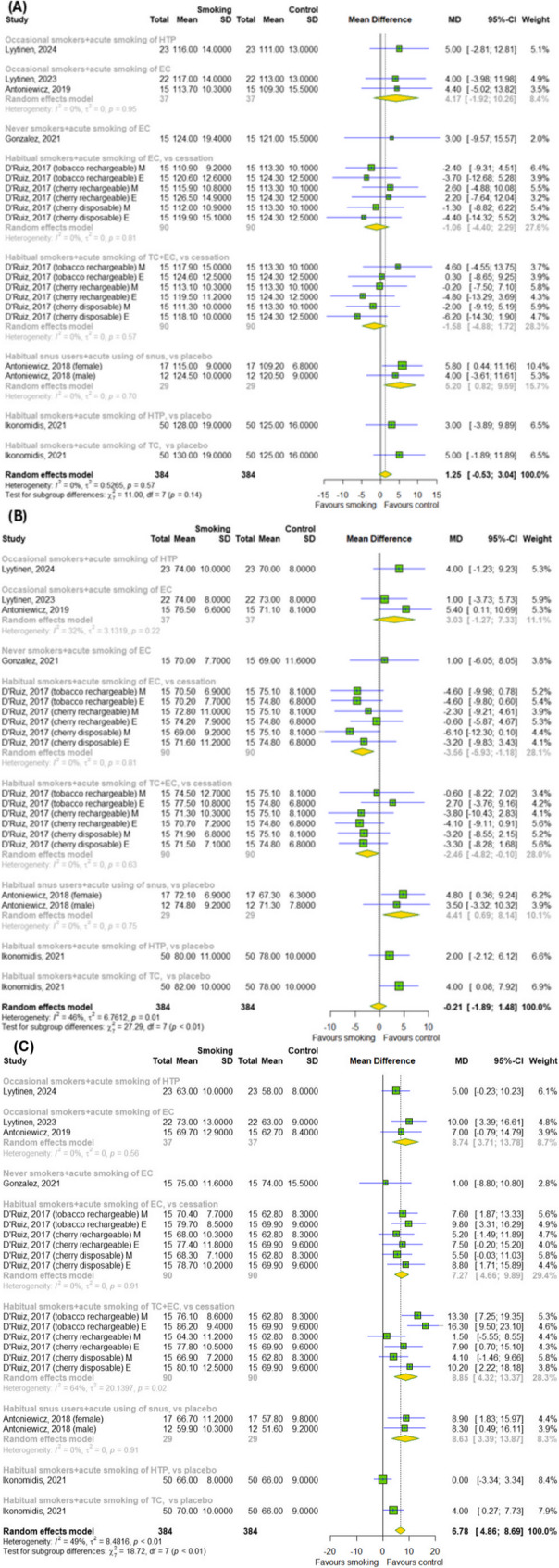
The effect of acute smoking on cardiovascular parameters (**A**) SBP, (**B**) DBP and (**C**) HRT. Results for continuous outcomes presented as the mean difference (MD) with 95% confidence intervals (95% CI). Effect sizes calculated by random effect model. I^2^ statistic: I^2^ < 40% may not be important; 30% < I^2^ < 60% moderate heterogeneity; 50% < I^2^ < 90% substantial heterogeneity; I^2^ > 75% considerable heterogeneity. The results at *p* < 0.05 were considered statistically significant. Abbreviations: *CI – confidence intervals; DBP – diastolic blood pressure; EC – e-cigarette; E – evening; HRT – heart rate; HTP – heated tobacco product; MD – mean difference; M – morning; SD- standard deviation; SBP – systolic blood pressure; TC – traditional cigarette*

We conducted a meta-analysis to evaluate the effect of acute smoking on cardiovascular parameters and pulmonary function. Regarding cardiovascular parameters, our meta-analysis showed that the overall effect of acute smoking (TCs, ECs, HTPs and snus) does not affect SBP and DBP, as shown in Fig. 2A-B (*p* > 0.05). Although we detected a significant difference between subgroups for the DBP (*p* = 0.0003). A meta-analysis identified a significant difference for the HRT (MD = 6.78; 95% CI [4.86; 8.69]); *p* < 0.0001; I^2^ = 49%) with difference between subgroups (*p* = 0.009), as shown in Fig. 2C.

**Fig. 3:**
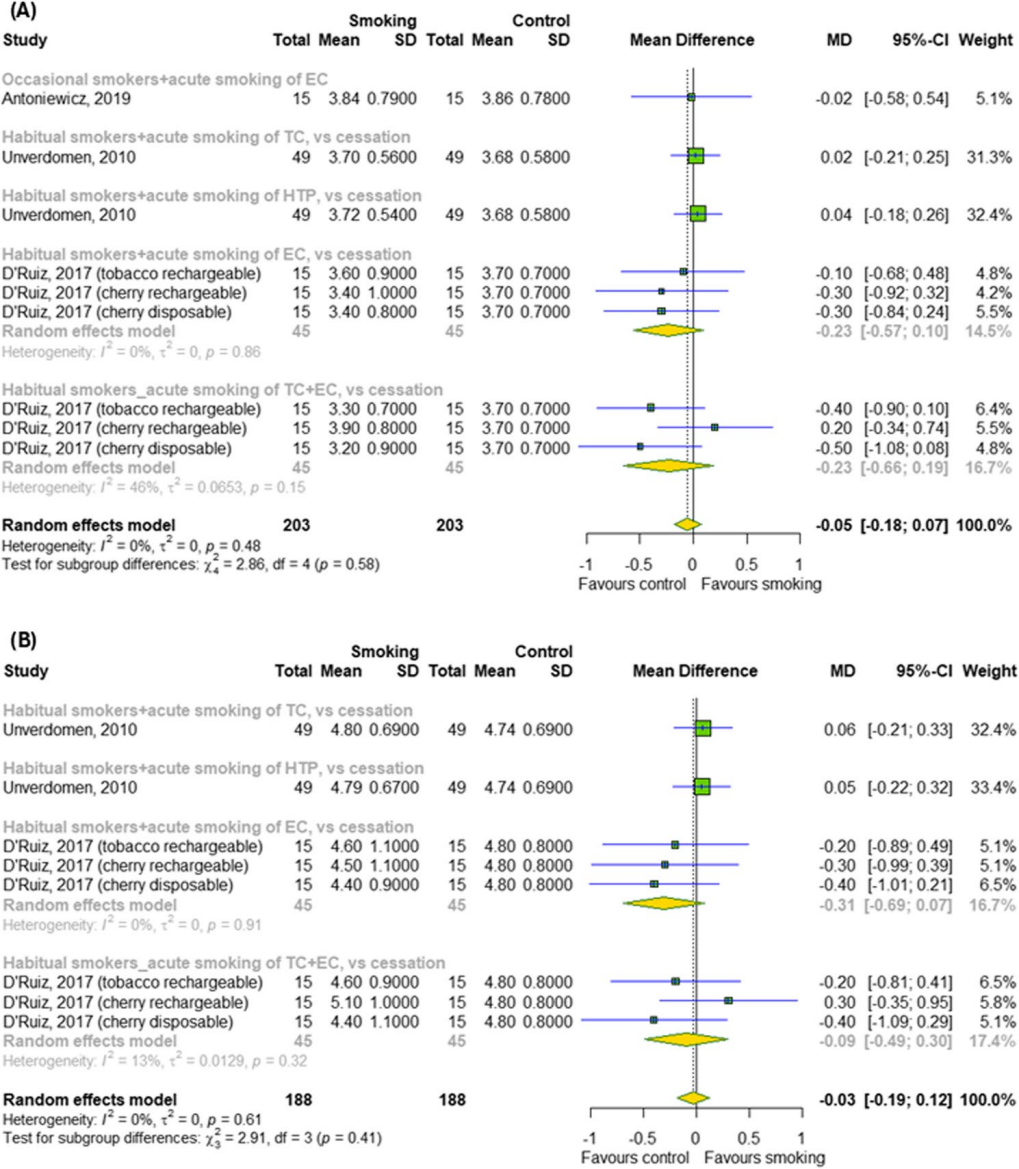
The effect of acute smoking on pulmonary function (**A**) FEV_1_ and (**B**) FVC. Results for continuous outcomes presented as the mean difference (MD) with 95% confidence intervals (95% CI). Effect sizes calculated by random effect model. I^2^ statistic: I^2^ < 40% may not be important; 30% < I^2^ < 60% moderate heterogeneity; 50% < I^2^ < 90% substantial heterogeneity; I^2^ > 75% considerable heterogeneity. The results at *p* < 0.05 were considered statistically significant. Abbreviations: *CI – confidence intervals; EC – e-cigarette; FEV*_*1*_ *– forced expiratory volume in one second; FVC – forced vital capacity; HTP – heated tobacco product; MD – mean difference; SD- standard deviation; TC – traditional cigarette*

Our meta-analysis showed no significant difference between the smoking (TCs, ECs and HTPs) and control groups for the pulmonary function, such as FEV_1_ and FVC without difference between subgroups (*p* > 0.05) (Fig. 3A-B).

### The effect of chronic smoking on metabolic parameters

**Fig. 4:**
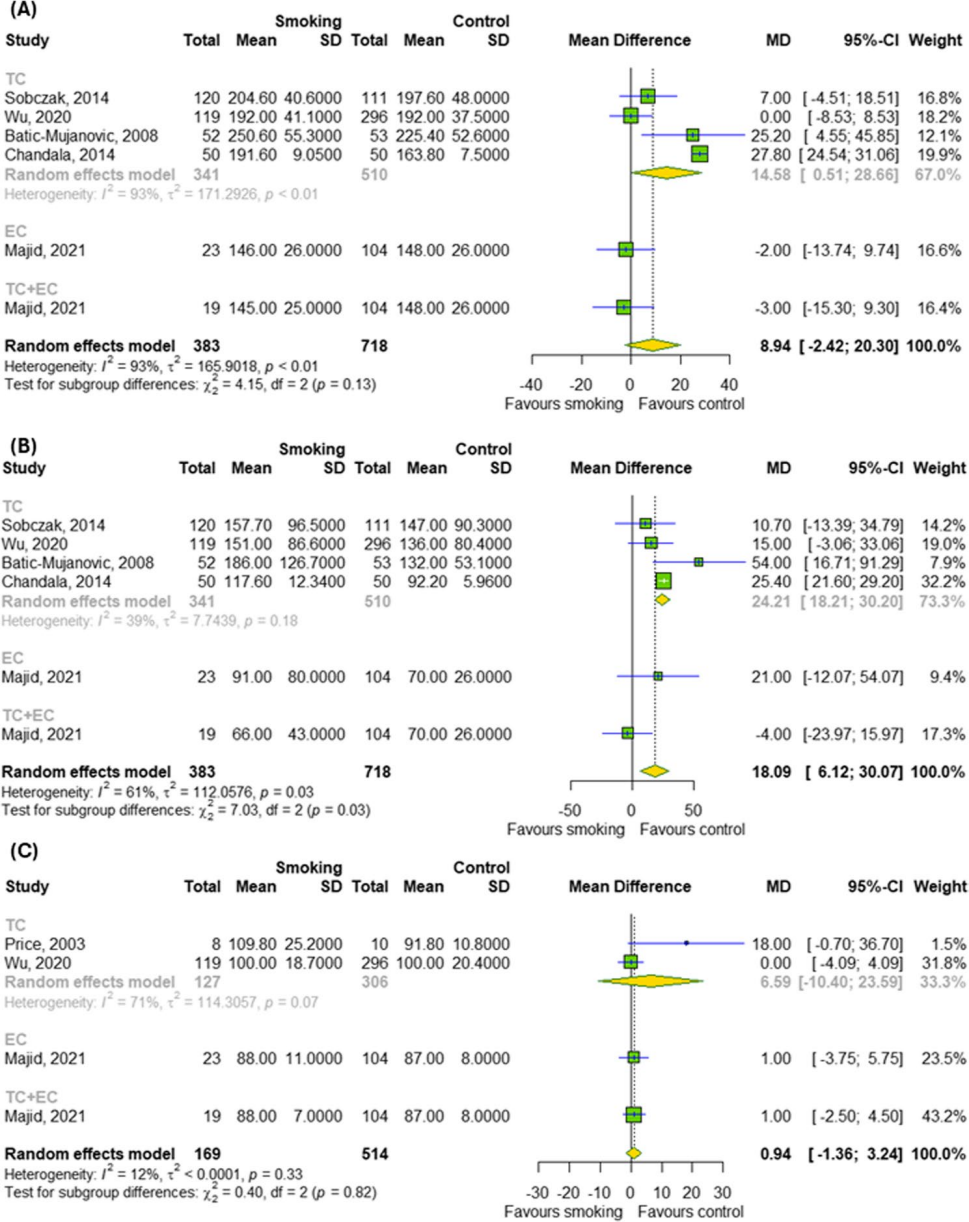
The effect of chronic smoking on (**A**) total cholesterol, (**B**) triglycerides and (**C**) glucose levels. Results for continuous outcomes presented as the mean difference (MD) with 95% confidence intervals (95% CI). Effect sizes calculated by random effect model. I^2^ statistic: I^2^ < 40% may not be important; 30% < I^2^ < 60% moderate heterogeneity; 50% < I^2^ < 90% substantial heterogeneity; I^2^ > 75% considerable heterogeneity. The results at *p* < 0.05 were considered statistically significant. Abbreviations: *CI – confidence intervals; EC – electronic cigarette; MD – mean difference; SD – standard deviation; TC – traditional cigarette*

In the next step, we examined the effect of chronic smoking on metabolic parameters, as showed in Fig. 4A-C. Among metabolic parameters (cholesterol, triglycerides and glucose levels), the meta-analysis found a significant effect of chronic smoking (TCs and ECs) only on triglycerides levels (MD = 18.09; 95% CI [6.12; 30.07]); *p* = 0.003; I^2^ = 61%) with differences between subgroups (*p* = 0.03).

### The effect of chronic smoking on cardiovascular parameters and pulmonary function

**Fig. 5:**
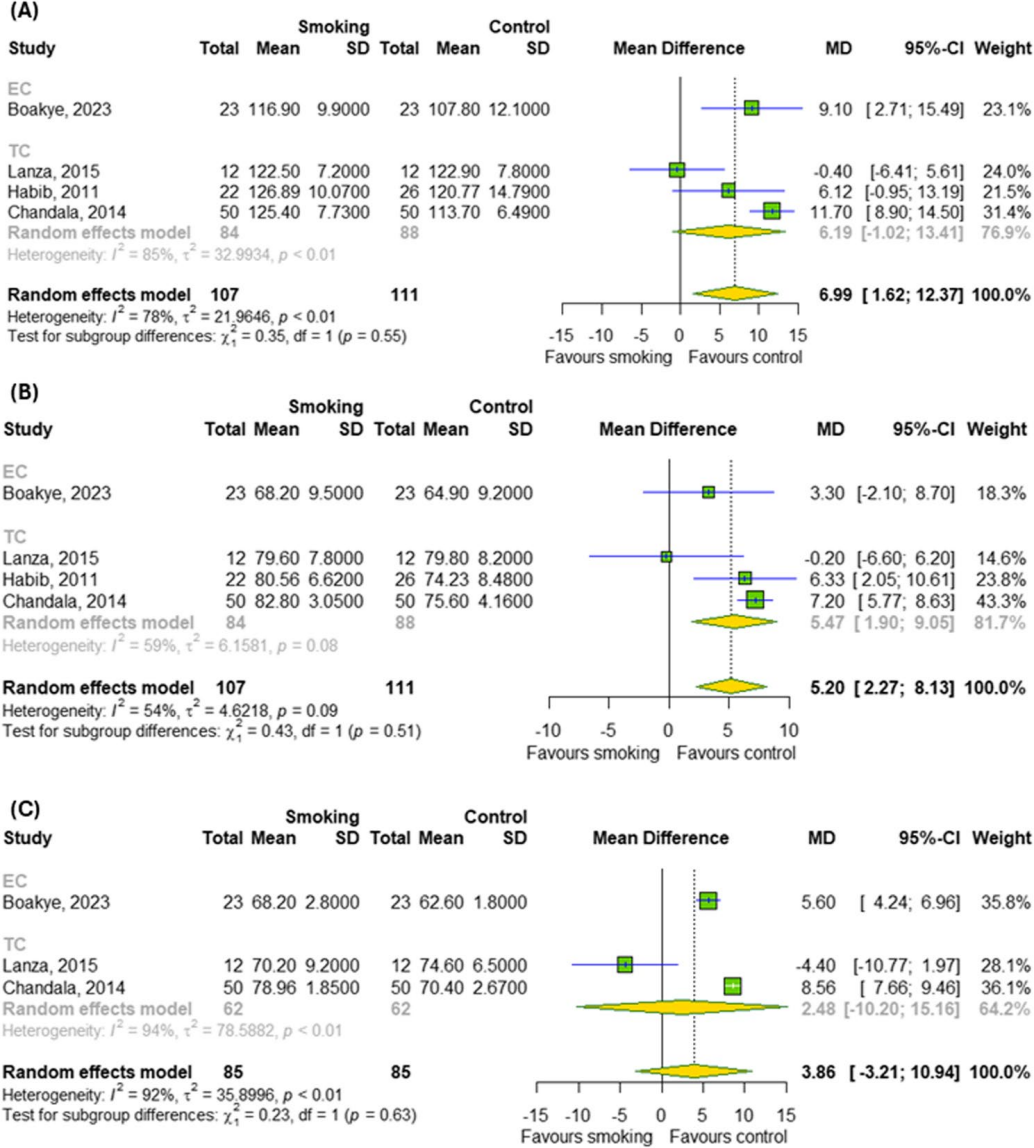
The effect of chronic smoking on cardiovascular parameters (**A**) SBP, (**B**) DBP and (**C**) HRT. Results for continuous outcomes presented as the mean difference (MD) with 95% confidence intervals (95% CI). Effect sizes calculated by random effect model. I^2^ statistic: I^2^ < 40% may not be important; 30% < I^2^ < 60% moderate heterogeneity; 50% < I^2^ < 90% substantial heterogeneity; I^2^ > 75% considerable heterogeneity. The results at *p* < 0.05 were considered statistically significant. Abbreviations: *CI – confidence intervals; DBP – diastolic blood pressure; EC – e-cigarette; HRT – heart rate; MD – mean difference; SBP – systolic blood pressure; SD- standard deviation; TC – traditional cigarette*

**Fig. 6:**
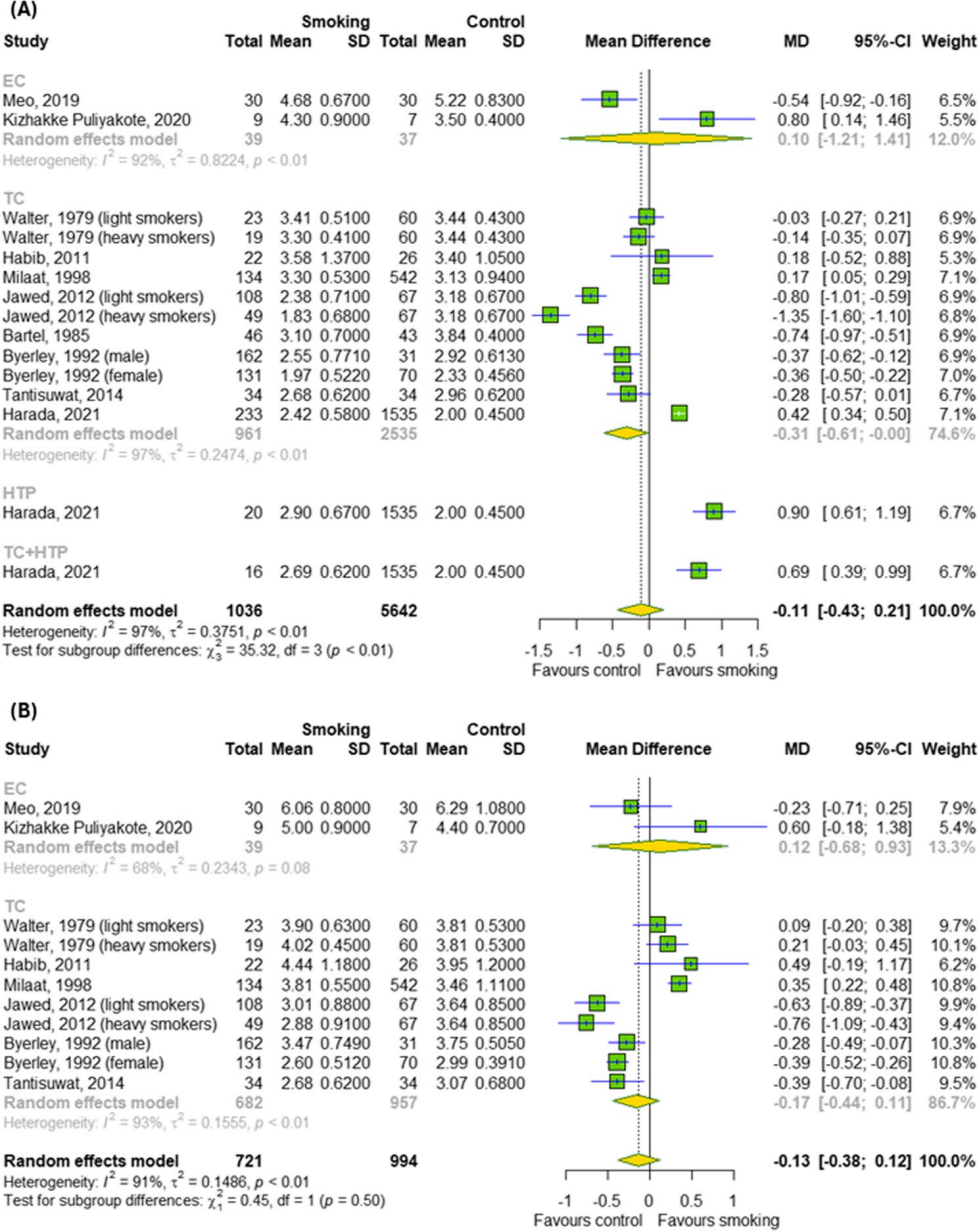
The effect of chronic smoking on pulmonary function (**A**) FEV_1_ and (**B**) FVC. Results for continuous outcomes presented as the mean difference (MD) with 95% confidence intervals (95% CI). Effect sizes calculated by random effect model. I^2^ statistic: I^2^ < 40% may not be important; 30% < I^2^ < 60% moderate heterogeneity; 50% < I^2^ < 90% substantial heterogeneity; I^2^ > 75% considerable heterogeneity. The results at *p* < 0.05 were considered statistically significant. Abbreviations: *CI – confidence intervals; EC – e-cigarette; FEV*_*1*_ *– forced expiratory volume in one second; FVC – forced vital capacity; HTP - heated tobacco products; MD – mean difference; SD- standard deviation; TC – traditional cigarette*

The impact of chronic smoking on cardiovascular parameters and pulmonary function was also investigated, as showed in Figs. 5A-C and 6A-B, respectively. Among the cardiovascular parameters (SBP, DBP and HRT), the meta-analysis overall detected a significant effect of chronic smoking (TCs and ECs) only on SBP (MD = 6.99; 95% CI [1.62; 12.37]); *p* = 0.01; I^2^ = 78%) and DBP (MD = 5.2; 95% CI [2.27; 8.13]); *p* = 0.0005; I^2^ = 54%) without differences between subgroups (*p* > 0.05). Among the pulmonary functions, meta-analysis didn’t show a significant effect of chronic smoking (TCs, ECs and HTPs) on FEV_1_ as well as FVC. In contrast, subgroup analysis indicated a difference between different types of cigarettes for FEV_1_ (*p* < 0.0001).

### The effect of chronic smoking on risk of cancer development

**Fig. 7:**
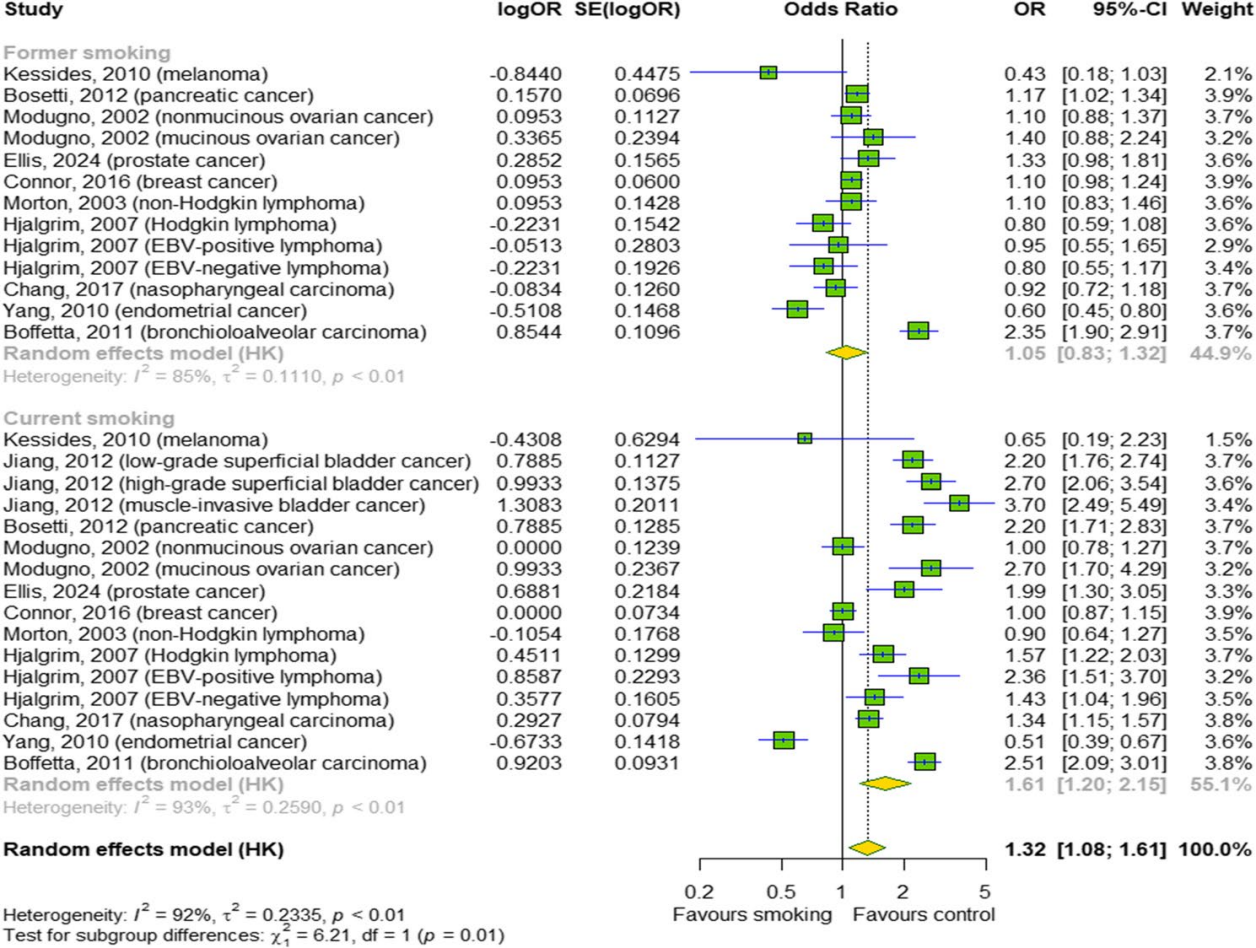
The effect of chronic smoking on the risk of cancer development. The cumulative effect measured based on odds ratio (OR) with 95% confidence intervals (95% CI). Effect sizes calculated by random effect model. I^2^ statistic: I^2^ < 40% may not be important; 30% < I^2^ < 60% moderate heterogeneity; 50% < I^2^ < 90% substantial heterogeneity; I^2^ > 75% considerable heterogeneity. The results at *p* < 0.05 were considered statistically significant. *Abbreviations: CI - confidence intervals; OR – odds ratio; SE – standard error*

In the final stage, we examined the effect of chronic smoking of traditional cigarettes on the risk of developing cancer (Fig. 7). Meta-analysis shows greater odds of cancer with chronic smoking by 32% (OR = 1.32; 95% CI [1.08; 1.61]); *p* = 0.009; I^2^ = 92%) with differences between subgroups (*p* = 0.01). We have shown that former smokers have a 5% higher chance of developing cancer, while current smokers have an increased chance by as much as 61%.

### Publication of bias

We created funnel plots (*Additional file 6*) and performed an Egger’s regression test to estimate publication bias for the outcomes under investigation. The results showed that there was no evidence of publication bias for the association between smoking and outcomes, such as: SBP for acute smoking (*p* = 0.21), DBP for acute smoking (*p* = 0.12), FEV_1_ for acute smoking (*p* = 0.07), FVC for acute smoking (*p* = 0.08), total cholesterol level (*p* = 0.06), triglycerides level (*p* = 0.42), glucose level (*p* = 0.07), SBP for chronic smoking (*p* = 0.25), DBP for chronic smoking (*p* = 0.11), HRT for chronic smoking (*p* = 0.28), FEV_1_ for chronic smoking (*p* = 0.17), FVC for chronic smoking (*p* = 0.86) and OR of association between smoking and cancers (*p* = 0.78). However, publication bias occur in HRT for acute smoking (*p* = 0.007).

## Discussion

To the best of our knowledge, this is the first meta-analysis examining the impact of various tobacco products, including conventional TCs and alternative products: ECs, HTPs and snus, on basic cardiovascular (SBP, DBP, HRT), metabolic (TRI, CHOL, GLU) and respiratory (FVC, FEV_1_) parameters, as well as overall risk of cancer development. This meta-analysis shows that acute exposure to analyzed tobacco products (TCs, ECs, HTP and snus) significantly affects heart rate, but not systolic and diastolic blood pressure. Furthermore, chronic exposure to analyzed tobacco products significantly affects systolic and diastolic blood pressure, but not heart rate. Moreover, we found that chronic exposure to analyzed tobacco products significantly affects triglycerides level, but not total cholesterol and glucose levels. In addition, there was a slight decline on respiratory parameters, FVC and FEV_1_, due to acute and chronic smoking of TCs, ECs and HTP compared to smoking cessation or never smoking. However such changes were not statistically significant. Finally, results of our meta-analysis determined greater odds of cancer among current and former TCs smokers than in never-smokers. The high observed heterogeneity (I² = 92%) of studies for cancer risk was anticipated given the variability in cancer etiology, latency periods, exposure duration, smoking intensity, and inclusion of multiple cancer sites with potentially different or even inverse associations.

A meta-analysis of 23 population-based studies presented by *Linneberg et al.* showed that current smokers had 2.40 mmHg and 1.93 mmHg lower systolic and diastolic blood pressure and 1.93 bpm higher resting heart rate compared to never-smokers, which is in opposite to results of our meta-analysis. However, authors found that 1 cigarette/day increase in smoking intensity increases SBP by 0.08 mmHg, DBP by 0.05 mmHg and resting HRT by 0.21 bpm [76]. An analysis of 12 studies by *Kundu et al.* found increase in SBP (MD = 12.856; *p* < 0.01), DBP (MD = 7.676; *p* < 0.01) and HRT (MD = 11.329; *p* < 0.01) after acute exposure to e-cigarettes compared to non-use. Moreover, authors found no significant differences in BP and resting HRT in current EC-users compared to non-users and a reduction in HRT (MD= -2.608; *p* < 0.01) and DBP (MD= -3.226; *p* < 0.01) compared with current TC-users. As authors suggested, despite some evidence for endothelial dysfunction associated with ECs use, short-to-medium-term switching from TCs to ECs may improve blood pressure, especially among females and young people [77]. Decline in blood pressure after switching from long-term TCs to ECs use has been related to reduction of the negative impact of tobacco smoking on acute vasopressor and tachycardia responses, as well as arterial stiffness [78]. Another meta-analysis of 14 studies by *Skotsimara et al.* determined that acute exposure to e-cigarettes increased HRT by 2.27 bpm, DBP by 2.01 mmHg and SBP by 2.02 mmHg compared to non-users. Moreover, switching from TCs to ECs leaded to reduction of SBP by 7.00 mmHg and DBP by 3.65 mmHg [79]. This findings suggested that using od ECs as an alternative to conventional cigarettes may reduce negative cardiovascular outcomes associated with smoking. However, such issue should be further investigated, due to some evidence on the association between ECs and the increased cardiovascular risk [80, 81]. In addition, this analysis focused only on the classic tobacco EC with rechargeable cartomizer (and nicotine) and our analysis included studies with any type of EC (with/without nicotine, any flavor and device type) [79]. A meta-analysis of 27 studies presented by *Siddiqi et al.* found that acute exposure to ECs significantly increased HRT (WMD = 0.76 bpm; *p* < 0.00001), SBP (WMD = 0.28 mmHg; *p* = 0.01) and DBP (WMD = 0.38 mmHg; *p* = 0.0006) compared to non-use. Moreover, increase in HRT has been observed for ECs with nicotine (WMD = 1.22 bpm; *p* < 0.00001), but not for ECs without nicotine. Similar results have been obtained for blood pressure, where increase in SBP and DBP was observed for ECs with nicotine (WMD = 0.51 mmHg; *p* = 0.001 for SBP; WMD = 0.62 mmHg; *p* < 0.0001 for DBP), but not for ECs without nicotine [82]. A pooled analysis of 15 studies presented by *Rahman et al.* showed increase in SBP (MD = 2.89; *p* < 0.001), DBP (MD = 3.10; *p* = 0.02) and HRT (MD = 3.13; *p* = 0.005) in ECs with nicotine users compared to non-users. Moreover, there was an increase in DBP (MD = 3.44; *p* = 0.01), but no in SBP in nicotine-free EC users compared to non-users. These results suggest that the effect of EC on cardiovascular parameters may depend on the composition and nicotine content of individual products [83]. *Barrientos et al.* meta-analysis of 13 RCTs showed 3.16 bpm reduction in HRT, but no differences in SBP and DBP in HTP smokers compared to TC smokers [84].

A meta-analysis of 169 studies presented by *van der Plas et al.* found 0.50 mmol/L higher triglycerides level in smokers compared to non-smokers, which is consistent with the results of our analysis [85]. Results of *Craig et al.* meta-analysis indicated 11.8% higher serum level of triglycerides and 3.7% lower level of total cholesterol in 8–19 years-old smokers compared to never-smokers. We also found higher triglycerides level, but no lower total cholesterol in smokers. Such differences in results may be associated with older age of the study participants included to our analysis [86]. A meta-analysis of 27 studies on the effect of smoking status on lipid profile by *Maeda et al.* found that smoking cessation did not significantly changed level of triglycerides and total cholesterol, which was potentially associated with remarkable heterogeneity of the studies [87]. A meta-analysis of 14 observational studies presented by *Kar et al.* found 0.61% higher level of HbA1c in smokers compared to non-smokers, but no significant difference in HbA1c in smokers compared to quitters. Furthermore, higher HbA1c levels indicate higher blood glucose levels in smokers. However, this study included patients with type 1 and type 2 diabetes, and our meta-analysis was based on healthy individuals. which may contribute to the significant difference in the obtained results [88].

The effect of smoking of tobacco products (compared to never smoking or smoking cessation) on the decline in respiratory parameters has been confirmed in several other studies. An analysis of 47 epidemiological studies on the effect of smoking status on respiratory parameters presented by *Lee and Fry* found that patients who continued to smoke had higher FEV_1_ decline (β = 40.8) compared to patients who quit smoking (β = 32.2; *p* < 0.001) and former smokers (β = 28.0; *p* < 0.001). However, such analysis included studies with healthy individuals, as well as patients with COPD, chronic bronchitis or emphysema, which were excluded from our analysis [89]. A study presented by *Honeycutt et al.* found that acute exposure to EC aerosol had no effect on FEV_1_ or slight decrease in FEV_1_, which returned to baseline level after 15 min, which is consistent with our results. In addition, this study showed that chronic EC use did not change FEV_1_ and FVC compared to non-users. However, 3 out of 8 included studies examined the effect of EC use on lung function among healthy individuals and patients with COPD and asthma [90]. A meta-analysis of 17 studies on acute respiratory response by *Laure et al.* found that immediate EC exposure (healthy and asthmatic individuals) did not significantly change any spirometry parameters [91]. A systematic review of 13 studies for chronic ECs use on pulmonary parameters by *Wasfi et al.* reported a mixed findings. Most of the studies reported no significant effect of chronic EC use on FEV_1_ and FVC compared to never-smokers or TC smokers. Only 3 cross-sectional studies indicated decline in FEV_1_ and FVC in chronic EC users compared to non-smokers and 2 studies reported increase in such parameters compared to TC use [92]. An meta-analysis of 24 studies measured BoPH (including pulmonary parameters) presented by *Braznell et al.* showed an decline in FEV_1_ in HTP users compared to abstinence. However, there was also an improvement in pulmonary parameters (including FEV_1_ and FVC) in HTP users compared to TCs users. However, this meta-analysis based mostly on tobacco industry-affiliated studies (13 out of 24 studies), with high risk of bias. Moreover, data have been analyzed only by effect direction plots, due to the quantity of different measured biomarkers, a paucity of studies and heterogeneity of the data [93].

Due to the fact that carcinogenic mechanism and risk factors differ substantially by organ, we discussed below the relationship between TCs smoking and different cancers. A meta-analysis of 40 studies by *Possenti et al.* confirmed direct association between smoking and nasopharyngeal cancer (61% higher risk for current smokers, 28% for former smokers and 62% for ever smokers) compared to never-smokers [94]. Another meta-analysis of 50 case-control and cohort studies by *Peng et al.* showed increased risk of breast cancer for smoking initiated at age ≤ 15 years (by 8%), at age 15–20 years (by 10%) and at age ≥ 20 years (by 8%) [95]. A meta-analysis of 50 studies presented by *Al-Fayez and El-Metwally* indicated that TCs smoking has an inverse association with prostate cancer incidence (16% lower risk), but 42% higher risk of death from prostate cancer compared to abstinence [96]. *Zhou et al.* found inverse association between smoking and the risk of endometrial cancer (16% lower risks for prospective and 27% lower risk for case-control studies) [97]. An analysis of 44 case-control and 8 cohort studies by *Zhao et al.* confirmed a non-linear dose–response relationship between TCs smoking and a risk of bladder cancer (3.9% higher risk for 1 more cigarette/day) [98]. Finally, an pooled analysis of 205 studies by *Rota et al.* confirmed a dose-response association between TCs and the risk of gastric cancer (53% higher risk for current smokers and 30% for former smokers) [99].

The molecular background associated with the negative health effects of conventional cigarettes smoking has been well-established. However, there is little known about such effect for alternative tobacco products. Therefore, on this paragraph we discussed the potential molecular mechanisms associated with the development of changes in the cardiovascular, metabolic and pulmonary parameters analyzed in this meta-analysis, as well as potential mechanism of carcinogenesis. Here, we mostly focused on the molecular activity of nicotine, the main component of such alternative tobacco products. However, we also pointed out some implication for another active compounds, if their effect have been well-established. The activation of sympathetic nervous system by nicotine results in an immediate and pronounced increase in the heart rate, contractility and cardiac output [100–103]. Acute exposure to cigarette smoke impairs endothelium-dependent relaxation of the brachial artery, due to low bioavailability of ^•^NO and formation of ONOO- [104]. In addition, carbon monoxide, has an impact on reduction in the blood’s oxygen-carrying capacity [105–107]. Moreover, in smokers endothelial dysfunction has been associated with increased activity of NADH oxidase, increased production of superoxide radical (O_2_^−^) and further lipids oxidation [108], activation of pro-inflammatory processes in the endothelial cells [109–112], decrease in nitric oxide signaling and promotion of vasoconstriction [113]. Activation of nAChRs by nicotine on islet cells, reduces insulin release from pancreatic β cells [114], but in skeletal muscles promotes insulin resistance [115, 116]. Moreover, components of tobacco smoke may have direct toxic effects on the pancreas and β-cell function [117]. In adipose tissue, nicotine may affects ineffective clearance of triglycerides. Furthermore, overproduction of ROS induces inhibition of insulin-mediated reduction of lipolysis. Activation of lipolysis induces free fatty acids delivery to the liver and skeletal muscles, resulting in the synthesis and secretion of very low-density lipoproteins (VLDL), rise in triglycerides and low-density lipoprotein (LDL) cholesterol [118]. Moreover, nicotine decline level of high-density lipoprotein (HDL) cholesterol and promotes production of more atherogenic lipid profile [119–122]. Cigarette smoke are a source of huge amount of oxidants. Moreover, toxic constituents of cigarette smoke induces production of oxidants by cells, which increases airway inflammation [123–125]. This leads to destruction of lung tissue and impairment of defense and repair mechanisms [123, 126–128]. Abnormal repair process and bronchiolar fibrosis is the result of activation of fibroblasts by factors released by infiltrating pro-inflammatory cells. Increased level of oxidants disrupt the oxidative-antioxidative balance and activate the transcription factors for pro-inflammatory mediators, which mediate prolonged lung inflammation. Oxidative stress activates inflammatory cells to increased secretion of proteases and inactivation of antiproteases, which leads to alveolar destruction of wall and increased secretion of mucous [129, 130]. Increased risk of tobacco-related cancers results directly from the activity of toxic components of tobacco products, as well as the activation of epigenetic changes by nicotine [131]. Nitrosamines via nAChRs [132–134] or β-adrenoreceptor (β-AR) [135, 136], as well as PAHs via aryl hydrocarbon receptor (AhR) [137, 138] induce cell proliferation and reduce apoptosis. Moreover, nitrosamines induce cell cycle progression [139] and malignant cells transformation [140]. Furthermore, nicotine by activation of DNA methyltransferases [141–143], histone methyltransferase [144] and regulation of miRNAs expression [145] leaded to induction of cell proliferation, tumour progression and metastasis.

We discussed above how using conventional and alternative tobacco products affects cardiovascular, metabolic and pulmonary parameters. In this paragraph we would like to summarize how disturbances in such parameters resulted in cardiovascular, metabolic and respiratory diseases development in the future. Recent data shows that an increase in SBP by 20 mmHg and DBP by 10 mmHg increased the risk of heart failure with reduced ejection fraction (HFrEF). In addition, an increase in SBP by 20 mmHg increased risk of heart failure with preserved ejection fraction (HFpEF). Furthermore, an increase in SBP by 10 mmHg and DBP by 5 mmHg increased risk of cardiovascular disease (CVD), coronary artery disease (CAD) and stroke [146]. Furthermore, increase in resting heart rate by 10 bpm increased risk of CAD, stroke, non-cardiovascular diseases and cancer [147]. Current epidemiological studies showed that increase in triglycerides level (≥ 150 mg/dL) increased risk of ischemic stroke [148], atherosclerotic cardiovascular disease (ASVD) [149, 150] and acute pancreatitis [151]. Moreover, increase in total cholesterol level (≥ 180 mg/dL) was positively associated with increased risk of ischemic heart disease, cerebrovascular disease (CEVD) [152], CHD and stroke [153]. High glucose level is positively associated with elevated level of triglycerides, LDL levels, triglycerides/HDL ratio and LDL/HDL ratio in diabetic patients [154]. In addition, chronic hyperglycemia leaded to microvascular complications, including nephropathy, retinopathy and neuropathy [155–157]. Abnormalities in early adulthood in FEV_1_ have been classified as a risk factor of COPD [158, 159], as well as a higher risk of early pulmonary and non-pulmonary comorbidities and mortality [160, 161]. Moreover, decline in FVC has been characteristic for IPF [162], COPD [163], chronic bronchitis (CB) without airflow limitation [164] and a marked decline in FCV and FEV_1_ is characteristic during asthma exacerbation [165]. Furthermore, reduction in FEV_1_ and FVC (< 60%) is positively associated with increased risk of myocardial infarction (MI), ischemic stroke and heart failure [166].

This systematic review and meta-analysis had some limitations. There is a limited number of data associated with the tobacco heating products and snus, which make it impossible to provide in detail analysis of health-related consequences of their use. Moreover, many of the analyzed studies based on the self-reported data on the tobacco products use behaviors, which increased risk of response bias. Furthermore, there are significant differences in the chemical composition of specific tobacco products between manufacturers, which may impacted differences in their health effects. In addition, the variety in the brands, as well as nicotine and flavors content available around the world, limited investigation of the health effects to specific products, i.e. most frequent chosen by customers, available on the local/national market or characteristic for specific manufacturers, not for whole type of tobacco product. Furthermore, we excluded from analysis biomarkers of exposure (BoE) and potential harm (BoPH), which limits comparative interpretation between alternative products and TCs. Finally, this meta-analysis combined different study types (RCTs, cross-sectional, observational, cohort, case-control), which may have impact on the quality of the results and the risk of bias.

## Conclusions

Robust evidence supports adverse health effects of traditional cigarettes, whereas evidence for alternative products remains limited and heterogeneous. Therefore, longitudinal studies involving individuals exclusively using conventional and alternative tobacco products should be performed in the future.

## Supplementary Information

Supplementary Material 1.

Supplementary Material 2.

Supplementary Material 3.

Supplementary Material 4.

Supplementary Material 5.

Supplementary Material 6.

## Data Availability

All data generated or analyzed during this study are included in this published article and supplementary materials.

## Abbreviations

ACS: Acute coronary syndrome
AMD: Age–related macular degeneration
AMI: Acute myocardial infarction
ASVD: Atherosclerotic cardiovascular disease
BoE: Biomarker of exposure
BoPH: Biomarker of potential hazard
CAD: Coronary artery disease
CB: Chronic bronchitis
CEVD: Cerebrovascular disease
CHD: Coronary heart disease
CHOL: Total cholesterol
CI: Confidence interval
CO: Carbon monoxide
COPD: Chronic obstructive pulmonary disease
CVD: Cardiovascular disease
DBP: Diastolic blood pressure
EC: Electronic cigarette
ENDS: Electronic nicotine delivery system
EU: European Union
FEV_1_: Forced expiratory volume in one second
FVC: Forced vital capacity
GLU: Glucose
HDL: High–density lipoprotein
HF: Heart failure
HFpEF: Heart failure with preserved ejection fraction
HFrEF: Heart failure with reduced ejection fraction
HPHC: Hazardous and potentially hazardous constituent
HR: Hazard rate
HRT: Heart rate
HTP: Heated tobacco product
IARC: International Agency for Research on Cancer
IPF: Idiopathic pulmonary fibrosis
LDL: Low–density lipoprotein
MD: Mean difference
MI: Myocardial infarction
NNK − 4: (Methylnitrosamino)–1–(3–pyridyl)–1–butanone
NSCLC: Non–small cell lung cancer
OR: Odds ratio
OS: Oxidative stress
PAD: Peripheral arterial disease
PAH: Polycyclic aromatic hydrocarbon
pMD: Pooled mean difference
RA: Rheumatoid arthritis
ROS: Reactive oxygen species
RR: Relative risk
SBP: Systolic blood pressure
SD: Standard deviation
SE: Standard error
sSAD: Spirometric small airway dysfunction
TC: Traditional cigarette
TRI: Triglycerides
TSNA: Tobacco–specific nitrosamine
US: United States
VLDL: Very low–density lipoprotein
VOC: Volatile organic compound
WHO: World Health Organization
WMD: Weighted mean difference

## References

[1] WHO global report on trends in prevalence of tobacco use 2000–2030. Available from: https://www.who.int/publications/i/item/9789240088283. Cited 2025 May 6.

[2] WHO report on the global tobacco epidemic, 2023: protect people from tobacco smoke. Available from: https://www.who.int/publications/i/item/9789240077164. Cited 2025 May 6.

[3] Harris JE. Cigarette smoke components and disease: cigarette smoke is more than a triad of tar, nicotine, and carbon monoxide. Smok Tob control Monogr. 1996;7:59–75.

[4] Rodgman A, Smith CJ, Perfetti TA. The composition of cigarette smoke: a retrospective, with emphasis on polycyclic components. Hum Exp Toxicol. 2000;19(10):10. 10.1191/096032700701546514. 10.1191/096032700701546514 11211997

[5] Soleimani F, Dobaradaran S, De-la-Torre GE, Schmidt TC, Saeedi R. Content of toxic components of cigarette, cigarette smoke vs cigarette butts: a comprehensive systematic review. Sci Total Environ. 2022;813:152667. 10.1016/j.scitotenv.2021.152667. 34963586 10.1016/j.scitotenv.2021.152667

[6] Smith CJ, Perfetti TA, Rumple MA, Rodgman A, Doolittle DJ. IARC group 2A Carcinogens reported in cigarette mainstream smoke. Food Chem Toxicol. 2000;38(4):371–83. 10722891 10.1016/s0278-6915(99)00156-8

[7] Smith CJ, Perfetti TA, Rumple MA, Rodgman A, Doolittle DJ. IARC Group 2B carcinogens reported in cigarette mainstream smoke. Food Chem Toxicol. 2001;39(2):183–205. 10.1016/S0278-6915(00)00164-2. 11267712 10.1016/s0278-6915(00)00164-2

[8] Smith CJ, Livingston SD, Doolittle DJ. An international literature survey of IARC group I carcinogens reported in mainstream cigarette smoke. Food Chem Toxicol. 1997;35(10):1107–30. 10.1016/S0278-6915(97)00063-X. 9463546 10.1016/s0278-6915(97)00063-x

[9] Halliwell B, Poulsen HE, editors. Cigarette smoke and oxidative stress. Berlin: Springer; 2006. p. 407.

[10] Rogers TJ, Criner GJ, Cornwell WD. Smoking and lung inflammation. New York: Springer New York; 2013. Available from: 10.1007/978-1-4614-7351-0. Cited 2019 May 10.

[11] van der Vaart H. Acute effects of cigarette smoke on inflammation and oxidative stress: a review. Thorax. 2004;59(8):713–21. 10.1136/thx.2003.012468. 15282395 10.1136/thx.2003.012468 PMC1747102

[12] McKelvey K, Popova L, Kim M, Chaffee BW, Vijayaraghavan M, Ling P, et al. Heated tobacco products likely appeal to adolescents and young adults. Tob Control. 2018;27(Suppl 1):s41–7. 10.1136/tobaccocontrol-2018-054596. 30352843 10.1136/tobaccocontrol-2018-054596 PMC6252490

[13] Geiss O, Bianchi I, Barahona F, Barrero-Moreno J. Characterisation of mainstream and passive vapours emitted by selected electronic cigarettes. Int J Hyg Environ Health. 2015;218(1):169–80. 10.1016/j.ijheh.2014.10.001. 25455424 10.1016/j.ijheh.2014.10.001

[14] Alawsi F, Nour R, Prabhu S. Are e-cigarettes a gateway to smoking or a pathway to quitting? BDJ. 2015;219(3):111–5. 10.1038/sj.bdj.2015.591. 26271862 10.1038/sj.bdj.2015.591

[15] Goniewicz ML, Kuma T, Gawron M, Knysak J, Kosmider L. Nicotine Levels in electronic cigarettes. Nicotine Tob Res. 2013;15(1):158–66. 10.1093/ntr/nts103. 22529223 10.1093/ntr/nts103

[16] Lawler TS, Stanfill SB, Tran HT, Lee GE, Chen PX, Kimbrell JB, et al. Chemical analysis of snus products from the United States and Northern Europe. PLoS ONE. 2020;15(1):e0227837. 10.1371/journal.pone.0227837. PubMed PMID: 31940415; PubMed Central PMCID: PMC6961908. 31940415 10.1371/journal.pone.0227837 PMC6961908

[17] Chen AX, Akmam Morsed F, Cheah NP. A simple method to simultaneously determine the level of nicotine, glycerol, propylene glycol, and triacetin in heated tobacco products by gas chromatography–flame-ionization detection. J AOAC Int. 2022;105(1):46–53. 10.1093/jaoacint/qsab140. 34648035 10.1093/jaoacint/qsab140

[18] Wasowicz A, Feleszko W, Goniewicz ML. E-Cigarette use among children and young people: the need for regulation. Expert Rev Respir Med. 2015;9(5):507–9. 10.1586/17476348.2015.1077120. 26290119 10.1586/17476348.2015.1077120

[19] Hahn J, Monakhova YB, Hengen J, Kohl-Himmelseher M, Sch?ssler J, Hahn H, et al. Electronic cigarettes: overview of chemical composition and exposure estimation. Tob Induc Dis. 2014;12(1). 10.1186/s12971-014-0023-6. 10.1186/s12971-014-0023-6 PMC4304610 25620905

[20] Hildick-Smith GJ, Pesko MF, Shearer L, Hughes JM, Chang J, Loughlin GM, et al. A practitioner’s guide to electronic cigarettes in the adolescent population. J Adolesc Health. 2015;57(6):574–9. 10.1016/j.jadohealth.2015.07.020. 26422289 10.1016/j.jadohealth.2015.07.020

[21] Farsalinos KE, Yannovits N, Sarri T, Voudris V, Poulas K, Leischow SJ. Carbonyl emissions from a novel heated tobacco product (IQOS): comparison with an e-cigarette and a tobacco cigarette: Carbonyl emissions in heated tobacco product. Addiction. 2018;113(11):2099–106. 10.1111/add.14365. 29920842 10.1111/add.14365

[22] Leigh NJ, Palumbo MN, Marino AM, O’Connor RJ, Goniewicz ML. Tobacco-specific nitrosamines (TSNA) in heated tobacco product IQOS. Tob Control. 2018;27(Suppl 1):s37–8. 10.1136/tobaccocontrol-2018-054318. 30242043 10.1136/tobaccocontrol-2018-054318 PMC6252482

[23] Gometz ED. Health effects of smoking and the benefits of quitting. Virtual Mentor. 2011;13(1):31. 23121814 10.1001/virtualmentor.2011.13.1.cprl1-1101

[24] Talhout R, Schulz T, Florek E, van Benthem J, Wester P, Opperhuizen A. Hazardous compounds in tobacco smoke. Int J Environ Res Public Health. 2011;8(2):613–28. 10.3390/ijerph8020613. PubMed PMID: 21556207; PubMed Central PMCID: PMC3084482. 21556207 10.3390/ijerph8020613 PMC3084482

[25] Sussan TE, Gajghate S, Thimmulappa RK, Ma J, Kim JH, Sudini K, et al. Exposure to electronic cigarettes impairs pulmonary anti-bacterial and anti-viral defenses in a mouse model. PLoS ONE. 2015;10(2):e0116861. 25651083 10.1371/journal.pone.0116861 PMC4317176

[26] Dautzenberg B. Tobacco-related diseases. Rev Prat. 2004;54(17):1877–82. PubMed PMID: 15655911. 15655911

[27] He H, Pan Z, Wu J, Hu C, Bai L, Lyu J. Health effects of tobacco at the global, regional, and national levels: results from the 2019 global burden of disease study. Nicotine Tob Res. 2022;24(6):864–70. 10.1093/ntr/ntab265. PubMed PMID: 34928373. 34928373 10.1093/ntr/ntab265

[28] West R. Tobacco smoking: Health impact, prevalence, correlates and interventions. Psychol Health. 2017;32(8):1018–36. 10.1080/08870446. .2017.1325890 PubMed PMID: 28553727; PubMed Central PMCID: PMC5490618. 28553727 10.1080/08870446.2017.1325890 PMC5490618

[29] Risk Factors: Tobacco - NCI [cgvArticle]. 2015. Located at: nciglobal,ncienterprise. Available from: https://www.cancer.gov/about-cancer/causes-prevention/risk/tobacco. Cited 2024 Nov 7.

[30] Forum NCP, Services B on HC, Medicine I of Tobacco use and cancer. In: Reducing tobacco-related cancer incidence and mortality: workshop summary. National Academies Press (US); 2013. Available from: https://www.ncbi.nlm.nih.gov/books/NBK206898/. Cited 2024 Nov 7. 24901191

[31] CDCTobaccoFree. Centers for Disease Control and Prevention. Health Effects of Cigarette Smoking. 2021. Available from: https://www.cdc.gov/tobacco/data_statistics/fact_sheets/health_effects/effects_cig_smoking/index.htm. Cited 2022 Apr 11.

[32] Is nicotine addictive? | National Institute on Drug Abuse (NIDA). Available from: https://nida.nih.gov/publications/research-reports/tobacco-nicotine-e-cigarettes/nicotine-addictive. Cited 2025 May 6.

[33] Page MJ, McKenzie JE, Bossuyt PM, Boutron I, Hoffmann TC, Mulrow CD, et al. The PRISMA 2020 statement: an updated guideline for reporting systematic reviews. BMJ. 2021;n71. 10.1136/bmj.n71. 10.1136/bmj.n71 PMC8005924 33782057

[34] Higgins JPT, Altman DG, Gotzsche PC, Juni P, Moher D, Oxman AD, et al. The cochrane collaboration’s tool for assessing risk of bias in randomised trials. BMJ. 2011;343(oct18 2):d5928–5928. 10.1136/bmj.d5928. 22008217 10.1136/bmj.d5928 PMC3196245

[35] Ottawa Hospital Research Institute. Available from: https://www.ohri.ca/programs/clinical_epidemiology/oxford.asp. Cited 2025 Jun 9.

[36] Downes MJ, Brennan ML, Williams HC, Dean RS. Development of a critical appraisal tool to assess the quality of cross-sectional studies (AXIS). BMJ Open. 2016;6(12):e011458. 10.1136/bmjopen-2016-011458. 27932337 10.1136/bmjopen-2016-011458 PMC5168618

[37] Deeks JJ, Higgins JP, Altman DG. Analysing data and undertaking meta-analyses. In: Higgins JP, Green S, editors. Cochrane handbook for systematic reviews of interventions. Chichester: John Wiley & Sons, Ltd; 2008. p. 243–96. 10.1002/9780470712184.ch9.

[38] Gonzalez JE, Cooke WH. Acute effects of electronic cigarettes on arterial pressure and peripheral sympathetic activity in young nonsmokers. Am J Physiol Heart Circ Physiol. 2021;320(1):H248–55. 10.1152/ajpheart.00448.2020. PubMed PMID: 33164580. 33164580 10.1152/ajpheart.00448.2020

[39] D’Ruiz CD, O’Connell G, Graff DW, Yan XS. Measurement of cardiovascular and pulmonary function endpoints and other physiological effects following partial or complete substitution of cigarettes with electronic cigarettes in adult smokers. Regul Toxicol Pharmacol. 2017;87:36–53. 10.1016/j.yrtph.2017.05.002. PubMed PMID: 28476553. 28476553 10.1016/j.yrtph.2017.05.002

[40] Price TB, Krishnan-Sarin S, Rothman DL. Smoking impairs muscle recovery from exercise. Am J Physiol Endocrinol Metab. 2003;285(1):E116–122. 10.1152/ajpendo.00543.2002. PubMed PMID: 12637259. 12637259 10.1152/ajpendo.00543.2002

[41] Boakye E, Uddin SMI, Osuji N, Meinert J, Obisesan OH, Mirbolouk M, et al. Examining the association of habitual e-cigarette use with inflammation and endothelial dysfunction in young adults: The VAPORS-Endothelial function study. Tob Induc Dis. 2023;21:75. 10.18332/tid/162327. PubMed PMID: 37305426; PubMed Central PMCID: PMC10257221. 37305426 10.18332/tid/162327 PMC10257221

[42] Wu P, Li W, Cai X, Yan H, Chen M, for Alzheimer’s Disease Neuroimaging Initiative. Associations of cigarette smoking with memory decline and neurodegeneration among cognitively normal older individuals. Neurosci Lett. 2020;714:134563. 10.1016/j.neulet.2019.134563. PubMed PMID: 31678372. 31678372 10.1016/j.neulet.2019.134563

[43] Kessides MC, Wheless L, Hoffman-Bolton J, Clipp S, Alani RM, Alberg AJ. Cigarette smoking and malignant melanoma: a case-control study. J Am Acad Dermatol. 2011;64(1):84–90. 10.1016/j.jaad.2010.01.041. 20334951 10.1016/j.jaad.2010.01.041 PMC2924442

[44] Jiang X, Castelao JE, Yuan JM, Stern MC, Conti DV, Cortessis VK, et al. Cigarette smoking and subtypes of bladder cancer. Int J Cancer. 2012;130(4):896–901. 10.1002/ijc.26068. PubMed PMID: 21412765; PubMed Central PMCID: PMC3210924. 21412765 10.1002/ijc.26068 PMC3210924

[45] Modugno F, Ness RB, Cottreau CM. Cigarette smoking and the risk of mucinous and nonmucinous epithelial ovarian cancer. Epidemiology. 2002;13(4):467–71. 10.1097/00001648-200207000-00016. PubMed PMID: 12094103. 12094103 10.1097/00001648-200207000-00016

[46] Ellis ET, Fairman BJ, Stahr SD, Bensen JT, Mohler JL, Song L, et al. Cigarette smoking and prostate cancer aggressiveness among African and European American men. Cancer Causes Control. 2024;35(9):1259–69. 10.1007/s10552-024-01883-3. PubMed PMID: 38758522; PubMed Central PMCID: PMC11377453. 38758522 10.1007/s10552-024-01883-3 PMC11377453

[47] Connor AE, Baumgartner KB, Baumgartner RN, Pinkston CM, Boone SD, John EM, et al. Cigarette smoking and breast cancer risk in hispanic and non-hispanic white women: the breast cancer health disparities study. J Womens Health (Larchmt). 2016;25(3):299–310. 10.1089/jwh.2015.5502. PubMed PMID: 26682495; PubMed Central PMCID: PMC4790199. 26682495 10.1089/jwh.2015.5502 PMC4790199

[48] Morton LM, Holford TR, Leaderer B, Boyle P, Zahm SH, Zhang Y, et al. Cigarette smoking and risk of non-Hodgkin lymphoma subtypes among women. Br J Cancer. 2003;89(11):2087–92. 10.1038/sj.bjc.6601388. PubMed PMID: 14647142; PubMed Central PMCID: PMC2376853. 14647142 10.1038/sj.bjc.6601388 PMC2376853

[49] Boffetta P, Jayaprakash V, Yang P, Asomaning K, Muscat JE, Schwartz AG, et al. Tobacco smoking as a risk factor of bronchioloalveolar carcinoma of the lung: pooled analysis of seven case-control studies in the International Lung Cancer Consortium (ILCCO). Cancer Causes Control. 2011;22(1):73–9. 10.1007/s10552-010-9676-5. PubMed PMID: 21072579; PubMed Central PMCID: PMC3002160. 21072579 10.1007/s10552-010-9676-5 PMC3002160

[50] Majid S, Keith RJ, Fetterman JL, Weisbrod RM, Nystoriak J, Wilson T, et al. Lipid profiles in users of combustible and electronic cigarettes. Vasc Med. 2021;26(5):483–8. 10.1177/1358863X211009313. PubMed PMID: 34013801; PubMed Central PMCID: PMC10026074. 34013801 10.1177/1358863X211009313 PMC10026074

[51] Kizhakke Puliyakote AS, Elliott AR, Sá RC, Anderson KM, Crotty Alexander LE, Hopkins SR. Vaping disrupts ventilation-perfusion matching in asymptomatic users. J Appl Physiol (1985). 2021;130(2):308–17. 10.1152/japplphysiol.00709. .2020 PubMed PMID: 33180648; PubMed Central PMCID: PMC7948111. 33180648 10.1152/japplphysiol.00709.2020 PMC7948111

[52] Chen CL, Tang JS, Li PC, Chou PL. Immediate effects of smoking on cardiorespiratory responses during dynamic exercise: arm vs. leg ergometry. Front Physiol. 2015;6:376. 10.3389/fphys.2015.00376. PubMed PMID: 26696905; PubMed Central PMCID: PMC4674552. 26696905 10.3389/fphys.2015.00376 PMC4674552

[53] Chang ET, Liu Z, Hildesheim A, Liu Q, Cai Y, Zhang Z, et al. Active and passive smoking and risk of nasopharyngeal carcinoma: a population-based case-control study in Southern China. Am J Epidemiol. 2017;185(12):1272–80. 10.1093/aje/kwx018. PubMed PMID: 28459936; PubMed Central PMCID: PMC5860561. 28459936 10.1093/aje/kwx018 PMC5860561

[54] Meo SA, Ansary MA, Barayan FR, Almusallam AS, Almehaid AM, Alarifi NS, et al. Electronic cigarettes: impact on lung function and fractional exhaled nitric oxide among healthy adults. Am J Mens Health. 2019;13(1):1557988318806073. 10.1177/1557988318806073. PubMed PMID: 30318975; PubMed Central PMCID: PMC6771130. 30318975 10.1177/1557988318806073 PMC6771130

[55] Walter S, Nancy NR, Collier CR. Changes in the forced expiratory spirogram in young male smokers. Am Rev Respir Dis. 1979;119(5):717–24. 10.1164/arrd.1979.119.5.717. PubMed PMID: 453697. 453697 10.1164/arrd.1979.119.5.717

[56] Habib SS, Ahmed SM, Al Drees AM, Husain A. Effect of cigarette smoking on fractional exhaled nitric oxide in Saudi medical college students. J Pak Med Assoc. 2011;61(2):120–3. PubMed PMID: 21375156. 21375156

[57] Milaat WA, el-Ganai FM. Effects of cigarette smoking on lung function of Saudi students. Asia Pac J Public Health. 1998;10(1):39–42. 10.1177/101053959801000108 PubMed PMID: 10050206. 10050206 10.1177/101053959801000108

[58] Jawed S, Ejaz S, Rehman R. Influence of smoking on lung functions in young adults. J Pak Med Assoc. 2012;62(8):772–5. PubMed PMID: 23862247. 23862247

[59] Tantisuwat A, Thaveeratitham P. Effects of smoking on chest expansion, lung function, and respiratory muscle strength of youths. J Phys Ther Sci. 2014;26(2):167–70. 10.1589/jpts.26.167. PubMed PMID: 24648624; PubMed Central PMCID: PMC3944281. 24648624 10.1589/jpts.26.167 PMC3944281

[60] Harada S, Sata M, Matsumoto M, Iida M, Takeuchi A, Kato S, et al. Changes in smoking habits and behaviors following the introduction and spread of heated tobacco products in japan and its effect on FEV1 decline: a longitudinal cohort study. J Epidemiol. 2021;32. 10.2188/jea.JE20210075. 10.2188/jea.JE20210075 PMC8918621 34657910

[61] Chandala SR, Kilim SR. Plasma lipid profiles in chronic tobacco smokers and hypertensives. 2014. Available from: https://www.semanticscholar.org/paper/Plasma-Lipid-Profiles-In-Chronic-Tobacco-Smokers-Rao-Reddy/3d0e8e05d11caabffc7e3a37ff0f8a028cdc9c63. Cited 2025 Jun 28.

[62] Yang HP, Brinton LA, Platz EA, Lissowska J, Lacey JV, Sherman ME, et al. Active and passive cigarette smoking and the risk of endometrial cancer in Poland. Eur J Cancer. 2010;46(4):690–6. PubMed PMID: 20036529; PubMed Central PMCID: PMC2851155. 20036529 10.1016/j.ejca.2009.11.015 PMC2851155

[63] Hjalgrim H, Ekström-Smedby K, Rostgaard K, Amini RM, Molin D, Hamilton-Dutoit S, et al. Cigarette smoking and risk of Hodgkin lymphoma: a population-based case-control study. Cancer Epidemiol Biomarkers Prev. 2007;16(8):1561–6. 10.1158/1055-9965.EPI-07-0094. PubMed PMID: 17684129. 17684129 10.1158/1055-9965.EPI-07-0094

[64] Lanza GA, Spera FR, Villano A, Russo G, Di Franco A, Lamendola P, et al. Effect of smoking on endothelium-independent vasodilatation. Atherosclerosis. 2015;240(2):330–2. 03.041 PubMed PMID: 25875383. 25875383 10.1016/j.atherosclerosis.2015.03.041

[65] Batic-Mujanovic O, Beganlic A, Salihefendic N, Pranjic N, Kusljugic Z. Influence of smoking on serum lipid and lipoprotein levels among family medicine patients. Med Arh. 2008;62(5–6):264–7. PubMed PMID: 19469266. 19469266

[66] Ikonomidis I, Vlastos D, Kostelli G, Kourea K, Katogiannis K, Tsoumani M, et al. Differential effects of heat-not-burn and conventional cigarettes on coronary flow, myocardial and vascular function. Sci Rep. 2021;11(1):11808. 10.1038/s41598-021-91245-9. 34083663 10.1038/s41598-021-91245-9 PMC8175445

[67] Antoniewicz L, Brynedal A, Hedman L, Lundbäck M, Bosson JA. Acute effects of electronic cigarette inhalation on the vasculature and the conducting airways. Cardiovasc Toxicol. 2019;19(5):441–50. 10.1007/s12012-019-09516-x. PubMed PMID: 30963443; PubMed Central PMCID: PMC6746878. 30963443 10.1007/s12012-019-09516-x PMC6746878

[68] Antoniewicz L, Novo M, Bosson J, Lundbäck M. Brief exposure to Swedish snus causes divergent vascular responses in healthy male and female volunteers. PLoS ONE. 2018;13(4):e0195493. 10.1371/journal.pone.0195493. PubMed PMID: 29668699; PubMed Central PMCID: PMC5905986. 29668699 10.1371/journal.pone.0195493 PMC5905986

[69] Lyytinen G, Brynedal A, Anesäter E, Antoniewicz L, Blomberg A, Wallén H, et al. Electronic cigarette vaping with nicotine causes increased thrombogenicity and impaired microvascular function in healthy volunteers: a randomised clinical trial. Cardiovasc Toxicol. 2023;23(7–8):255–64. 10.1007/s12012-023-09802-9. PubMed PMID: 37548804; PubMed Central PMCID: PMC10435650. 37548804 10.1007/s12012-023-09802-9 PMC10435650

[70] Lyytinen G, Melnikov G, Brynedal A, Anesäter E, Antoniewicz L, Blomberg A, et al. Use of heated tobacco products (IQOS) causes an acute increase in arterial stiffness and platelet thrombus formation. Atherosclerosis. 2024;390. 10.1016/j.atherosclerosis.2023.117335. PubMed PMID: 37872010. 10.1016/j.atherosclerosis.2023.117335 37872010

[71] Sobczak A, Prokopowicz A, Radek M, Szula M, Zaciera M, Kurek J, et al. Tobacco smoking decreases plasma concentration of the emerging cardiovascular risk marker, L-homoarginine. Circ J. 2014;78(5):1254–8. 10.1253/circj.cj-13-1334. PubMed PMID: 24583919. 24583919 10.1253/circj.cj-13-1334

[72] Barter SJ, Cunningham DA, Lavender JP, Gibellino F, Connellan SJ, Pride NB. Abnormal ventilation scans in middle-aged smokers. Comparison with tests of overall lung function. Am Rev Respir Dis. 1985;132(1):148–51. 10.1164/arrd.1985.132.1.148. PubMed PMID: 4014860. 4014860 10.1164/arrd.1985.132.1.148

[73] Unverdorben M, Mostert A, Munjal S, van der Bijl A, Potgieter L, Venter C, et al. Acute effects of cigarette smoking on pulmonary function. Regul Toxicol Pharmacol. 2010;57(2):241–6. 10.1016/j.yrtph.2009.12.013. 20233598 10.1016/j.yrtph.2009.12.013

[74] Byerley DM, Weitz CA, Richards F. Smoking and pulmonary function in five Solomon Island populations. Am J Phys Anthropol. 1992;89(1):11–7. 10.1002/ajpa.1330890103. PubMed PMID: 1530058. 1530058 10.1002/ajpa.1330890103

[75] Bosetti C, Lucenteforte E, Silverman DT, Petersen G, Bracci PM, Ji BT, et al. Cigarette smoking and pancreatic cancer: an analysis from the International Pancreatic Cancer Case-Control Consortium (Panc4). Ann Oncol. 2012;23(7):1880–8. 10.1093/annonc/mdr541. PubMed PMID: 22104574; PubMed Central PMCID: PMC3387822. 22104574 10.1093/annonc/mdr541 PMC3387822

[76] Linneberg A, Jacobsen RK, Skaaby T, Taylor AE, Fluharty ME, Jeppesen JL, et al. Effect of smoking on blood pressure and resting heart rate: a mendelian randomisation meta-analysis in the carta consortium. Circ Cardiovasc Genet. 2015;8(6):832–41. 10.1161/CIRCGENETICS.115.001225 PubMed PMID: 26538566; PubMed Central PMCID: PMC4684098. 26538566 10.1161/CIRCGENETICS.115.001225 PMC4684098

[77] Kundu A, Feore A, Sanchez S, Abu-Zarour N, Sutton M, Sachdeva K, et al. Cardiovascular health effects of vaping e-cigarettes: a systematic review and meta-analysis. Heart. 2025;111(13):599–608. 10.1136/heartjnl-2024-325030. PubMed PMID: 40010935. 40010935 10.1136/heartjnl-2024-325030

[78] Hahad O, Kuntic M, Kuntic I, Daiber A, Münzel T. Tobacco smoking and vascular biology and function: evidence from human studies. Pflugers Arch. 2023;475(7):797–805. 10.1007/s00424-023-02805-z PubMed PMID: 36961561; PubMed Central PMCID: PMC10264470. 36961561 10.1007/s00424-023-02805-z PMC10264470

[79] Skotsimara G, Antonopoulos AS, Oikonomou E, Siasos G, Ioakeimidis N, Tsalamandris S, et al. Cardiovascular effects of electronic cigarettes: A systematic review and meta-analysis. Eur J Prev Cardiol. 2019;26(11):1219–28. 10.1177/2047487319832975 PubMed PMID: 30823865. 30823865 10.1177/2047487319832975

[80] Alzahrani T, Pena I, Temesgen N, Glantz SA. Association between electronic cigarette use and myocardial infarction. Am J Prev Med. 2018;55(4):455–61. 10.1016/j.amepre.2018.05.004. PubMed PMID: 30166079; PubMed Central PMCID: PMC6208321. 30166079 10.1016/j.amepre.2018.05.004 PMC6208321

[81] Glantz SA, Nguyen N, Oliveira da Silva AL. Population-based disease odds for e-cigarettes and dual use versus cigarettes. NEJM Evid. 2024;3(3):EVIDoa2300229. 10.1056/EVIDoa2300229. 38411454 10.1056/EVIDoa2300229 PMC11562742

[82] Siddiqi TJ, Rashid AM, Siddiqi AK, Anwer A, Usman MS, Sakhi H, et al. Association of Electronic Cigarette Exposure on Cardiovascular Health: A Systematic Review and Meta-Analysis. Curr Probl Cardiol. 2023;48(9):101748. 10.1016/j.cpcardiol.2023.101748. PubMed PMID: 37088177. 37088177 10.1016/j.cpcardiol.2023.101748

[83] Rahman A, Alqaisi S, Alzakhari R, Saith S. Characterization and summarization of the impact of electronic cigarettes on the cardiovascular system: a systematic review and meta-analysis. Cureus 15(5):e39528. 10.7759/cureus.39528 PubMed PMID: 37366450; PubMed Central PMCID: PMC10290866. 10.7759/cureus.39528 PMC10290866 37366450

[84] Barrientos M, Reano JD, Arimado R, Castillo R. PS-C12-1: heated tobacco products vs traditional tobacco cigarettes effects on blood pressure and other cardiovascular risk predictors among smokers. A meta-analysis. J Hypertens. 2023;41(Suppl 1):e218. 10.1097/01.hjh.0000914920.14657.44.

[85] van der Plas A, Antunes M, Pouly S, de La Bourdonnaye G, Hankins M, Heremans A. Meta-analysis of the effects of smoking and smoking cessation on triglyceride levels. Toxicol Rep. 2023;10:367–75. 10.1016/j.toxrep.2023.03.001. PubMed PMID: 36926662; PubMed Central PMCID: PMC10011683. 36926662 10.1016/j.toxrep.2023.03.001 PMC10011683

[86] Craig WY, Palomaki GE, Johnson AM, Haddow JE. Cigarette smoking-associated changes in blood lipid and lipoprotein levels in the 8- to 19-year-old age group: a meta-analysis. Pediatrics. 1990;85(2):155–8. PubMed PMID: 2136949. 2136949

[87] Maeda K, Noguchi Y, Fukui T. The effects of cessation from cigarette smoking on the lipid and lipoprotein profiles: a meta-analysis. Prev Med. 2003;37(4):283–90. 10.1016/S0091-7435(03)00110-5. 14507483 10.1016/s0091-7435(03)00110-5

[88] Kar D, Gillies C, Zaccardi F, Webb D, Seidu S, Tesfaye S, et al. Relationship of cardiometabolic parameters in non-smokers, current smokers, and quitters in diabetes: a systematic review and meta-analysis. Cardiovasc Diabetol. 2016;15(1):158. 10.1186/s12933-016-0475-5. 27881170 10.1186/s12933-016-0475-5 PMC5121966

[89] Lee PN, Fry JS. Systematic review of the evidence relating FEV1 decline to giving up smoking. BMC Med. 2010;8:84. 10.1186/1741-7015-8-84. PubMed PMID: 21156048; PubMed Central PMCID: PMC3017006. 21156048 10.1186/1741-7015-8-84 PMC3017006

[90] Honeycutt L, Huerne K, Miller A, Wennberg E, Filion KB, Grad R, et al. A systematic review of the effects of e-cigarette use on lung function. NPJ Prim Care Respir Med. 2022;32(1):45. 10.1038/s41533-022-00311-w. 36273009 10.1038/s41533-022-00311-w PMC9588082

[91] Larue F, Tasbih T, Ribeiro PAB, Lavoie KL, Dolan E, Bacon SL. Immediate physiological effects of acute electronic cigarette use in humans: a systematic review and meta-analysis. Respir Med. 2021;190:106684. 10.1016/j.rmed.2021.106684. 34808583 10.1016/j.rmed.2021.106684

[92] Wasfi RA, Bang F, de Groh M, Champagne A, Han A, Lang JJ, et al. Chronic health effects associated with electronic cigarette use: a systematic review. Front Public Health. 2022;10:959622. 10.3389/fpubh.2022.959622. PubMed PMID: 36276349; PubMed Central PMCID: PMC9584749. 36276349 10.3389/fpubh.2022.959622 PMC9584749

[93] Braznell S, Dance S, Hartmann-Boyce J, Gilmore A. Impact of heated tobacco products on biomarkers of potential harm and adverse events: a systematic review and meta-analysis. Tob Control. 2025;tc-2024-059000. 10.1136/tc-2024-059000. 40300839 10.1136/tc-2024-059000 PMC13217111

[94] PossentiI, Martini A, Bagnardi V, Specchia C, Odone A, Smits LJM et al. Association between cigarette smoking and nasopharyngeal cancer risk: a meta-analysis. Rhinology. 2025;63(1)13–21. 10.4193/Rhin24.265. 10.4193/Rhin24.265 39440663

[95] Peng G, Lee G, Kim S, Chen QY, Park Y, Keum N. Meta-analysis of smoking and breast cancer risk: by age of smoking initiation. Breast Cancer. 2025;32(5):905–16. 10.1007/s12282-025-01715-5. 40461913 10.1007/s12282-025-01715-5

[96] Al-Fayez S, El-Metwally A. Cigarette smoking and prostate cancer: A systematic review and meta-analysis of prospective cohort studies. Tob Induc Dis. 2023;21:19. 10.18332/tid/157231. PubMed PMID: 36762260; PubMed Central PMCID: PMC9900478. 36762260 10.18332/tid/157231 PMC9900478

[97] Zhou B, Yang L, Sun Q, Cong R, Gu H, Tang N, et al. Cigarette smoking and the risk of endometrial cancer: a meta-analysis. Am J Med. 2008;121(6):501–e5083. 10.1016/j.amjmed.2008.01.044. PubMed PMID: 18501231. 18501231 10.1016/j.amjmed.2008.01.044

[98] Zhao X, Wang yuanli, Liang C. Cigarette smoking and risk of bladder cancer: a dose–response meta-analysis. Int Urol Nephrol. 2022;54(6):1169–85. 10.1007/s11255-022-03173-w. 35332429 10.1007/s11255-022-03173-w

[99] Rota M, Possenti I, Valsassina V, Santucci C, Bagnardi V, Corrao G, et al. Dose-response association between cigarette smoking and gastric cancer risk: a systematic review and meta-analysis. Gastric Cancer. 2024;27(2):197–209. 10.1007/s10120-023-01459-1. PubMed PMID: 38231449. 38231449 10.1007/s10120-023-01459-1

[100] Haass M, Kübler W. Nicotine and sympathetic neurotransmission. Cardiovasc Drug Ther. 1997;10(6):657–65. 10.1007/BF00053022. 10.1007/BF00053022 9110108

[101] Klein LW. Systemic and coronary hemodynamic effects of tobacco products on the cardiovascular system and potential pathophysiologic mechanisms. Cardiol Rev. 2022;30(4):188–96. 10.1097/CRD.0000000000000395 PubMed PMID: 34001689. 34001689 10.1097/CRD.0000000000000395

[102] Fernandez CJ, Hanna FWF, Pacak K, Nazari MA. Catecholamines and blood pressure regulation. In: Endocrine Hypertension. Academic Press; 2023. pp. 19–34. Available from: https://www.sciencedirect.com/science/chapter/edited-volume/abs/pii/B9780323961202000108. 10.1016/B978-0-323-96120-2.00010-8.

[103] Motiejunaite J, Amar L, Vidal-Petiot E. Adrenergic receptors and cardiovascular effects of catecholamines. Ann Endocrinol (Paris). 2021;82(3–4):193–7. 10.1016/j.ando.2020.03.012. PubMed PMID: 32473788. 32473788 10.1016/j.ando.2020.03.012

[104] Sarabi M, Lind L. Short-term effects of smoking and nicotine chewing gum on endothelium-dependent vasodilation in young healthy habitual smokers. J Cardiovasc Pharmacol. 2000;35(3):451–6. 10.1097/00005344-200003000-00016. PubMed PMID: 10710132. 10710132 10.1097/00005344-200003000-00016

[105] Papathanasiou G, Georgakopoulos D, Georgoudis G, Spyropoulos P, Perrea D, Evangelou A. Effects of chronic smoking on exercise tolerance and on heart rate-systolic blood pressure product in young healthy adults. Eur J Cardiovasc Prev Rehabil. 2007;14(5):646–52. 10.1097/HJR.0b013e3280ecfe2c. PubMed PMID: 17925623. 17925623 10.1097/HJR.0b013e3280ecfe2c

[106] Bernaards CM, Twisk JWR, Van Mechelen W, Snel J, Kemper HCG. A longitudinal study on smoking in relationship to fitness and heart rate response. Med Sci Sports Exerc. 2003;35(5):793–800. 10.1249/01.MSS.0000064955.31005.E0 PubMed PMID: 12750589. 12750589 10.1249/01.MSS.0000064955.31005.E0

[107] Zevin S, Saunders S, Gourlay SG, Jacob P, Benowitz NL. Cardiovascular effects of carbon monoxide and cigarette smoking. J Am Coll Cardiol. 2001;38(6):1633–8. 10.1016/s0735-1097(01)01616-3. PubMed PMID: 11704374. 11704374 10.1016/s0735-1097(01)01616-3

[108] Kiowski W, Linder L, Stoschitzky K, Pfisterer M, Burckhardt D, Burkart F, et al. Diminished vascular response to inhibition of endothelium-derived nitric oxide and enhanced vasoconstriction to exogenously administered endothelin-1 in clinically healthy smokers. Circulation. 1994;90(1):27–34. 10.1161/01.cir.90.1.27. PubMed PMID: 8026008. 8026008 10.1161/01.cir.90.1.27

[109] Mazzone A, Cusa C, Mazzucchelli I, Vezzoli M, Ottini E, Ghio S, et al. Cigarette smoking and hypertension influence nitric oxide release and plasma levels of adhesion molecules. Clin Chem Lab Med. 2001;39(9):822–6. 10.1515/CCLM.2001.136 PubMed PMID: 11601680. 11601680 10.1515/CCLM.2001.136

[110] Bermudez EA, Rifai N, Buring JE, Manson JE, Ridker PM. Relation between markers of systemic vascular inflammation and smoking in women. Am J Cardiol. 2002;89(9):1117–9. 10.1016/s0002-9149(02)02284-1. PubMed PMID: 11988205. 11988205 10.1016/s0002-9149(02)02284-1

[111] van der Vaart H. Acute effects of cigarette smoke on inflammation and oxidative stress: a review. Thorax. 2004;59(8):8. 10.1136/thx.2003.012468. 15282395 10.1136/thx.2003.012468 PMC1747102

[112] Reilly M, Delanty N, Lawson JA, FitzGerald GA. Modulation of oxidant stress in vivo in chronic cigarette smokers. Circulation. 1996;94(1):19–25. 10.1161/01.cir.94.1.19. PubMed PMID: 8964113. 8964113 10.1161/01.cir.94.1.19

[113] Messner B, Bernhard D. Smoking and cardiovascular disease: mechanisms of endothelial dysfunction and early atherogenesis. Arterioscler Thromb Vasc Biol. 2014;34(3):509–15. 10.1161/ATVBAHA.113.300156. PubMed PMID: 24554606. 24554606 10.1161/ATVBAHA.113.300156

[114] Molina J, Rodriguez-Diaz R, Fachado A, Jacques-Silva MC, Berggren PO, Caicedo A. Control of insulin secretion by cholinergic signaling in the human pancreatic islet. Diabetes. 2014;63(8):2714–26. 10.2337/db13-1371. 24658304 10.2337/db13-1371 PMC4113066

[115] Mashili F, Chibalin AV, Krook A, Zierath JR. Constitutive STAT3 phosphorylation contributes to skeletal muscle insulin resistance in type 2 diabetes. Diabetes. 2013;62(2):457–65. 10.2337/db12-0337. 23043161 10.2337/db12-0337 PMC3554355

[116] Gupta D, Lacayo AA, Greene SM, Leahy JL, Jetton TL. β-Cell mass restoration by α7 nicotinic acetylcholine receptor activation. J Biol Chem. 2018;293(52):20295–306. 10.1074/jbc.RA118.004617. 30397183 10.1074/jbc.RA118.004617 PMC6311516

[117] Śliwińska-Mossoń M, Milnerowicz H. The impact of smoking on the development of diabetes and its complications. Diab Vasc Dis Res. 2017;14(4):265–76. 10.1177/1479164117701876 PubMed PMID: 28393534. 28393534 10.1177/1479164117701876

[118] Wu Y, Song P, Zhang W, Liu J, Dai X, Liu Z, et al. Activation of AMPKα2 in adipocytes is essential for nicotine-induced insulin resistance in vivo. Nat Med. 2015;21(4):373–82. 10.1038/nm.3826. 25799226 10.1038/nm.3826 PMC4390501

[119] Hasan HMA, Arhouma TA, Khanfar MMA, Azzam MA. Study the Relationship between the Nicotine and Lipid Profile with Some Hematology Parameters in Serum of Smoker and Non-Smoker Blood Samples. J Biosci Med. 2022;10(6):6. 10.4236/jbm.2022.106003.

[120] Gepner AD, Piper ME, Johnson HM, Fiore MC, Baker TB, Stein JH. Effects of smoking and smoking cessation on lipids and lipoproteins: outcomes from a randomized clinical trial. Am Heart J. 2011;161(1):145–51. 10.1016/j.ahj.2010.09.023. PubMed PMID: 21167347; PubMed Central PMCID: PMC3110741. 21167347 10.1016/j.ahj.2010.09.023 PMC3110741

[121] Campbell SC, Moffatt RJ, Stamford BA. Smoking and smoking cessation—The relationship between cardiovascular disease and lipoprotein metabolism: a review. Atherosclerosis. 2008;201(2):225–35. 10.1016/j.atherosclerosis.2008.04.046. 18565528 10.1016/j.atherosclerosis.2008.04.046

[122] Yamaguchi Y, Matsuno S, Kagota S, Haginaka J, Kunitomo M. Oxidants in cigarette smoke extract modify low-density lipoprotein in the plasma and facilitate atherogenesis in the aorta of Watanabe heritable hyperlipidemic rabbits. Atherosclerosis. 2001;156(1):109–17. 10.1016/s0021-9150(00)00637-7. PubMed PMID: 11369003. 11369003 10.1016/s0021-9150(00)00637-7

[123] Laniado-Laborín R, Smoking, Chronic Obstructive Pulmonary Disease (COPD). Parallel Epidemics of the 21st Century. Int J Environ Res Public Health. 2009;6(1):209–24. 10.3390/ijerph6010209. 19440278 10.3390/ijerph6010209 PMC2672326

[124] MacNee W. Pathogenesis of chronic obstructive pulmonary disease. Proc Am Thorac Soc. 2005;2(4):258–66. 10.1513/pats.200504-045SR. 16267346 10.1513/pats.200504-045SR PMC2713323

[125] Tamimi A, Serdarevic D, Hanania NA. The effects of cigarette smoke on airway inflammation in asthma and COPD: Therapeutic implications. Respir Med. 2012;106(3):319–28. 10.1016/j.rmed.2011.11.003. 22196881 10.1016/j.rmed.2011.11.003

[126] Tuder RM, Petrache I. Pathogenesis of chronic obstructive pulmonary disease. J Clin Invest. 2012;122(8):2749–55. 10.1172/JCI60324. 22850885 10.1172/JCI60324 PMC3408733

[127] Rabe KF, Hurd S, Anzueto A, Barnes PJ, Buist SA, Calverley P, et al. Global strategy for the diagnosis, management, and prevention of chronic obstructive pulmonary disease: GOLD executive summary. Am J Respir Crit Care Med. 2007;176(6):6. 10.1164/rccm.200703-456SO. 17507545 10.1164/rccm.200703-456SO

[128] McGuinness A, Sapey E. Oxidative stress in COPD: sources, markers, and potential mechanisms. J Clin Med. 2017;6(2):2. 10.3390/jcm6020021. 10.3390/jcm6020021 PMC5332925 28212273

[129] Van Eeden SF, Sin DD. Oxidative stress in chronic obstructive pulmonary disease: a lung and systemic process. Can Respir J. 2013;20(1):27–9. 23457671 10.1155/2013/509130 PMC3628643

[130] Hansen EC, Walters J, Wood Baker R. Explaining chronic obstructive pulmonary disease (COPD): perceptions of the role played by smoking: chronic obstructive pulmonary disease and cigarette smoking. Sociol Health Illn. 2007;29(5):730–49. 10.1111/j.1467-9566.2007.01013.x. 17714340 10.1111/j.1467-9566.2007.01013.x

[131] Kopa-Stojak PN, Pawliczak R. Comparison of carcinogenic potential of alternative tobacco products. A systematic review. Toxicol Mech Methods. 0(0):1–15. 10.1080/15376516.2025.2536664 PubMed PMID: 40741827. 10.1080/15376516.2025.2536664 40741827

[132] Jull BA, Plummer HK, Schuller HM. Nicotinic receptor-mediated activation by the tobacco-specific nitrosamine NNK of a Raf-1/MAP kinase pathway, resulting in phosphorylation of c-myc in human small cell lung carcinoma cells and pulmonary neuroendocrine cells. J Cancer Res Clin Oncol. 2001;127(12):707–17. 10.1007/s004320100289. PubMed PMID: 11768610. 11768610 10.1007/s004320100289 PMC12164663

[133] West KA, Brognard J, Clark AS, Linnoila IR, Yang X, Swain SM, et al. Rapid Akt activation by nicotine and a tobacco carcinogen modulates the phenotype of normal human airway epithelial cells. J Clin Invest. 2003;111(1):81–90. 10.1172/JCI200316147. PubMed PMID: 12511591; PubMed Central PMCID: PMC151834. 12511591 10.1172/JCI16147 PMC151834

[134] Tsurutani J, Castillo SS, Brognard J, Granville CA, Zhang C, Gills JJ, et al. Tobacco components stimulate Akt-dependent proliferation and NFkappaB-dependent survival in lung cancer cells. Carcinogenesis. 2005;26(7):1182–95. 10.1093/carcin/bgi072. PubMed PMID: 15790591. 15790591 10.1093/carcin/bgi072

[135] Schuller HM, Porter B, Riechert A. Beta-adrenergic modulation of NNK-induced lung carcinogenesis in hamsters. J Cancer Res Clin Oncol. 2000;126(11):624–30. 10.1007/pl00008474. PubMed PMID: 11079726. 11079726 10.1007/PL00008474 PMC12165142

[136] Schuller HM, Cekanova M. NNK-induced hamster lung adenocarcinomas over-express beta2-adrenergic and EGFR signaling pathways. Lung Cancer. 2005;49(1):35–45. 10.1016/j.lungcan.2004.12.012. PubMed PMID: 15949588. 15949588 10.1016/j.lungcan.2004.12.012

[137] Tannheimer SL, Ethier SP, Caldwell KK, Burchiel SW. Benzo[a]pyrene- and TCDD-induced alterations in tyrosine phosphorylation and insulin-like growth factor signaling pathways in the MCF-10A human mammary epithelial cell line. Carcinogenesis. 1998;19(7):1291–7. 10.1093/carcin/19.7.1291. PubMed PMID: 9683191. 9683191 10.1093/carcin/19.7.1291

[138] Bláha L, Kapplová P, Vondrácek J, Upham B, Machala M. Inhibition of gap-junctional intercellular communication by environmentally occurring polycyclic aromatic hydrocarbons. Toxicol Sci. 2002;65(1):43–51. 10.1093/toxsci/65.1.43. PubMed PMID: 11752684. 11752684 10.1093/toxsci/65.1.43

[139] Ho YS, Chen CH, Wang YJ, Pestell RG, Albanese C, Chen RJ, et al. Tobacco-specific carcinogen 4-(methylnitrosamino)-1-(3-pyridyl)-1-butanone (NNK) induces cell proliferation in normal human bronchial epithelial cells through NFkappaB activation and cyclin D1 up-regulation. Toxicol Appl Pharmacol. 2005;205(2):133–48. 10.1016/j.taap.2004.09.019. PubMed PMID: 15893541. 15893541 10.1016/j.taap.2004.09.019

[140] Jin Q, Menter DG, Mao L, Hong WK, Lee HY. Survivin expression in normal human bronchial epithelial cells: an early and critical step in tumorigenesis induced by tobacco exposure. Carcinogenesis. 2008;29(8):1614–22. 10.1093/carcin/bgm234. PubMed PMID: 18635526; PubMed Central PMCID: PMC2516487. 18635526 10.1093/carcin/bgm234 PMC2516487

[141] Satta R, Maloku E, Zhubi A, Pibiri F, Hajos M, Costa E, et al. Nicotine decreases DNA methyltransferase 1 expression and glutamic acid decarboxylase 67 promoter methylation in GABAergic interneurons. Proc Natl Acad Sci. 2008;105(42):42. 10.1073/pnas.0808699105 PMC2570996 18852456

[142] Di YP, Zhao J, Harper R. Cigarette smoke induces MUC5AC protein expression through the activation of Sp1. J Biol Chem. 2012;287(33):33. 10.1074/jbc.M111.334375. 10.1074/jbc.M111.334375 PMC3431669 22700966

[143] Jin T, Hao J, Fan D. Nicotine induces aberrant hypermethylation of tumor suppressor genes in pancreatic epithelial ductal cells. Biochem Biophys Res Commun. 2018;499(4):934–40. 10.1016/j.bbrc.2018.04.022. 29626481 10.1016/j.bbrc.2018.04.022

[144] Chang YW, Singh KP. Nicotine-induced oxidative stress contributes to EMT and stemness during neoplastic transformation through epigenetic modifications in human kidney epithelial cells. Toxicol Appl Pharmcol. 2019;374:65–76. 10.1016/j.taap.2019.04.023. 10.1016/j.taap.2019.04.023 31047982

[145] Lei Z, Xiaomin Y, He H, Jian C, Xiaowu X. Nicotine downregulates microRNA-200c to promote metastasis and the epithelial–mesenchymal transition in human colorectal cancer cells. J Cell Physiol. 2019;234(2):1369–79. 10.1002/jcp.26933. 30076725 10.1002/jcp.26933

[146] Malik R, Georgakis MK, Vujkovic M, Damrauer SM, Elliott P, Karhunen V, et al. Relationship between blood pressure and incident cardiovascular disease: linear and nonlinear mendelian randomization analyses. Hypertension. 2021;77(6):2004–13. 10.1161/HYPERTENSIONAHA.120.16534. 33813844 10.1161/HYPERTENSIONAHA.120.16534 PMC8115430

[147] Zhang D, Wang W, Li F. Association between resting heart rate and coronary artery disease, stroke, sudden death and noncardiovascular diseases: a meta-analysis. CMAJ. 2016;188(15):E384–92. 10.1503/cmaj.160050. PubMed PMID: 27551034; PubMed Central PMCID: PMC5056889. 27551034 10.1503/cmaj.160050 PMC5056889

[148] Di Angelantonio E, Sarwar N, Perry P, Kaptoge S, Ray K, Thompson A, et al. Major Lipids, Apolipoproteins, and Risk of Vascular Disease. JAMA. 2009;302(18):1993–2000. 10.1001/jama.2009.1619. PubMed PMID: 19903920; PubMed Central PMCID: PMC3284229. 19903920 10.1001/jama.2009.1619 PMC3284229

[149] Chapman MJ, Ginsberg HN, Amarenco P, Andreotti F, Borén J, Catapano AL, et al. Triglyceride-rich lipoproteins and high-density lipoprotein cholesterol in patients at high risk of cardiovascular disease: evidence and guidance for management. Eur Heart J. 2011;32(11):1345–61. 10.1093/eurheartj/ehr112. PubMed PMID: 21531743; PubMed Central PMCID: PMC3105250. 21531743 10.1093/eurheartj/ehr112 PMC3105250

[150] Anagnostis P, Rizos CV, Liamis G, Rallidis L, Skoumas I, Kolovou G, et al. Exploring the correlation between triglyceride levels and atherosclerotic cardiovascular disease prevalence in adults with familial hypercholesterolemia: Insights from a cross-sectional analysis in the HELLAS-FH registry. J Clin Lipidol. 2025;19(3):442–50. 10.1016/j.jacl.2024.12.017. PubMed PMID: 39893109. 39893109 10.1016/j.jacl.2024.12.017

[151] Hansen SEJ, Madsen CM, Varbo A, Nordestgaard BG. Low-Grade Inflammation in the Association between Mild-to-Moderate Hypertriglyceridemia and Risk of Acute Pancreatitis: A Study of More Than 115000 Individuals from the General Population. Clin Chem. 2019;65(2):321–32. 10.1373/clinchem.2018.294926. PubMed PMID: 30518661. 30518661 10.1373/clinchem.2018.294926

[152] Jeong S, Choi S, Kim K, Kim SM, Lee G, Park SY, et al. Effect of Change in Total Cholesterol Levels on Cardiovascular Disease Among Young Adults. J Am Heart Assoc. 2018;7(12):e008819. 10.1161/JAHA.118.008819. PubMed PMID: 29899019; PubMed Central PMCID: PMC6220545. 29899019 10.1161/JAHA.118.008819 PMC6220545

[153] Peters SAE, Singhateh Y, Mackay D, Huxley RR, Woodward M. Total cholesterol as a risk factor for coronary heart disease and stroke in women compared with men: a systematic review and meta-analysis. Atherosclerosis. 2016;248:123–31. 10.1016/j.atherosclerosis.2016.03.016. PubMed PMID: 27016614. 27016614 10.1016/j.atherosclerosis.2016.03.016

[154] Wang L, Yan N, Zhang M, Pan R, Dang Y, Niu Y. The association between blood glucose levels and lipids or lipid ratios in type 2 diabetes patients: a cross-sectional study. Front Endocrinol. 2022;13. 10.3389/fendo.2022.969080. 10.3389/fendo.2022.969080 PMC9485560 36147575

[155] Yue S, Zhang J, Wu J, Teng W, Liu L, Chen L. Use of the monocyte-to-lymphocyte ratio to predict diabetic retinopathy. Int J Environ Res Public Health. 2015;12(8):10009–19. 10.3390/ijerph120810009. PubMed PMID: 26308022; PubMed Central PMCID: PMC4555325. 26308022 10.3390/ijerph120810009 PMC4555325

[156] Caramori ML, Mauer M. Diabetes and nephropathy. Curr Opin Nephrol Hypertens. 2003;12(3):273–82. 10.1097/00041552-200305000-00008. PubMed PMID: 12698065. 12698065 10.1097/00041552-200305000-00008

[157] Papatheodorou K, Papanas N, Banach M, Papazoglou D, Edmonds M. Complications of diabetes 2016. J Diabetes Res. 2016;2016:6989453. 10.1155/2016/6989453. PubMed PMID: 27822482; PubMed Central PMCID: PMC5086373. 27822482 10.1155/2016/6989453 PMC5086373

[158] Vestbo J, Edwards LD, Scanlon PD, Yates JC, Agusti A, Bakke P, et al. Changes in forced expiratory volume in 1 second over time in COPD. N Engl J Med. 2011;365(13):1184–92. 10.1056/NEJMoa1105482. 21991892 10.1056/NEJMoa1105482

[159] Kakavas S, Kotsiou OS, Perlikos F, Mermiri M, Mavrovounis G, Gourgoulianis K, et al. Pulmonary function testing in COPD: looking beyond the curtain of FEV1. npj Prim Care Respir Med. 2021;31(1):23. 10.1038/s41533-021-00236-w. 33963190 10.1038/s41533-021-00236-w PMC8105397

[160] Lange P, Celli B, Agustí A, Boje Jensen G, Divo M, Faner R, et al. Lung-function trajectories leading to chronic obstructive pulmonary disease. N Engl J Med. 2015;373(2):111–22. 10.1056/NEJMoa1411532. PubMed PMID: 26154786. 26154786 10.1056/NEJMoa1411532

[161] Agustí A, Noell G, Brugada J, Faner R. Lung function in early adulthood and health in later life: a transgenerational cohort analysis. Lancet Respir Med. 2017;5(12):935–45. 10.1016/S2213-2600(17)30434-4. PubMed PMID: 29150410. 29150410 10.1016/S2213-2600(17)30434-4

[162] Lassenius MI, Toppila I, Pöntynen N, Kasslin L, Kaunisto J, Kilpeläinen M et al. Forced Vital Capacity (FVC) decline, mortality and healthcare resource utilization in idiopathic pulmonary fibrosis. Eur Clin Respir J. 7(1):1702618. 10.1080/20018525.2019.1702618. PubMed PMID: 32002175; PubMed Central PMCID: PMC6968594. 10.1080/20018525.2019.1702618 PMC6968594 32002175

[163] Kim NY, Kim DK, Park S, Hwang YI, Seo H, Park D, et al. Risk Factors of FEV1/FVC Decline in COPD Patients. J Korean Med Sci. 2024;40(6):e32. 10.3346/jkms.2025.40.e32. PubMed PMID: 39962940; PubMed Central PMCID: PMC11832881. 10.3346/jkms.2025.40.e32 PMC11832881 39962940

[164] Zheng J, Chen R, Sun Y, Wen FQ, Ma Q, Liu C, et al. Differential decline in FEV1 and FVC Across COPD GOLD grades and non-obstructed chronic bronchitis. Am J Respir Crit Care Med. 2025;211(Abstracts):A6185–6185. 10.1164/ajrccm.2025.211.Abstracts.A6185.

[165] Stephen M. The impact of asthma on lung function indices: clinical implications and management strategies. J Pulmonary Respiratory Med. 2024;14(4). 10.37421/2161-105X.2024.14.690.

[166] Rydell A, Janson C, Lisspers K, Lin YT, Ärnlöv J. FEV1 and FVC as robust risk factors for cardiovascular disease and mortality: Insights from a large population study. Respir Med. 2024;227. 10.1016/j.rmed.2024.107614. PubMed PMID: 38670319. 10.1016/j.rmed.2024.107614 38670319

