# Supplementary material for: Comparison of the health-related outcomes for traditional cigarettes, e-cigarettes, heat-not-burn cigarettes and snus: a systematic review and meta-analysis
Source: BMC Public Health. 2026 Mar 26;26:1458. doi: 10.1186/s12889-026-27067-z (PMC13141496; doi:10.1186/s12889-026-27067-z)
Supplement: Supplementary file 1 — Supplementary Material 1. [file 12889_2026_27067_MOESM1_ESM.docx]

**Table S1. Full search strategy for PubMed, EMBASE, Scopus, Web of Science and the Cochrane Database of Systematic Reviews.**

| **Database** | **Search terms** | **Search Results (July 15, 2025)** |
| --- | --- | --- |
| **PubMed** | (cigarette [tiab] OR "conventional cigarette" [tiab] OR ends [tiab] OR "electronic nicotine delivery system" [tiab] OR "electronic cigarette" [tiab] OR e-cigarette [tiab] OR "heat-not-burn product" [tiab] OR "heat-not-burn cigarette" [tiab] OR "tobacco heating product" [tiab] OR "heated tobacco product" OR snus [tiab] OR "Swedish snuff" [tiab] OR "oral nicotine pouches" [tiab] OR "nicotine pouches" [tiab]) AND (health [tiab] OR "health effect" [tiab] OR "health impact" [tiab])  Filters: Free full text, Full text, Adaptive Clinical Trial, Case Reports, Clinical Study, Clinical Trial, Controlled Clinical Trial, Equivalence Trial, Evaluation Study, Observational Study, Pragmatic Clinical Trial, Randomized Controlled Trial, English, Humans | 1,377 |
| **EMBASE** | ('cigarette'/exp OR 'cigarette' OR 'conventional cigarette'/exp OR 'conventional cigarette' OR 'electronic cigarette'/exp OR 'electronic cigarette' OR 'heated tobacco product'/exp OR 'heated tobacco product' OR 'heat not burn tobacco'/exp OR 'heat not burn tobacco' OR 'snus'/exp OR 'snus' OR 'nicotine pouch'/exp OR 'nicotine pouch') AND ('health impact'/exp OR 'health impact') OR 'health effect'/exp OR 'health effect')  Filters: 'human'/de AND 'article'/it | 1,944 |
| **Scopus** | **Search 1:**  (TITLE-ABS-KEY (cigarette) OR TITLE-ABS-KEY ("conventional cigarette") OR TITLE-ABS-KEY (ends) OR TITLE-ABS-KEY ("electronic nicotine delivery system") OR TITLE-ABS-KEY ("electronic cigarette") OR TITLE-ABS-KEY (e-cigarette) OR TITLE-ABS-KEY ( "heat-not-burn product" ) OR TITLE-ABS-KEY ("heat-not-burn cigarette") OR TITLE-ABS-KEY ("tobacco heating product") OR TITLE-ABS-KEY ("heated tobacco product") OR TITLE-ABS-KEY (snus) OR TITLE-ABS-KEY ("oral nicotine pouches") OR TITLE-ABS-KEY ("nicotine pouches") OR TITLE-ABS-KEY ("Swedish snuff") AND TITLE-ABS-KEY (health) OR TITLE-ABS-KEY ("health effect") OR TITLE-ABS-KEY ("health impact")) AND (LIMIT-TO (LANGUAGE , "English")) AND (LIMIT-TO (EXACTKEYWORD, "Human")) AND ( LIMIT-TO (DOCTYPE , "ar" )) AND ( LIMIT-TO ( SRCTYPE , "j" )) AND (LIMIT-TO (OA , "all" )) AND (LIMIT-TO (SUBJAREA , "MEDI" )) | 71,177 |
|  | **Search 2:**  (TITLE-ABS-KEY (cigarette  OR  "electronic cigarette"  OR  e-cigarette  OR  "heat-not-burn"  OR  "heated tobacco product"  OR  snus  OR  "oral nicotine pouches"  AND  health  AND effect  OR  health  AND  impact)  AND  (LIMIT-TO ( DOCTYPE ,  "ar" ))  AND  (LIMIT-TO (LANGUAGE ,  "English"))  AND  (LIMIT-TO (EXACTKEYWORD,  "Human"))  AND  (LIMIT-TO (OA ,  "all" )) | 2,834 |
| **Web of Science** | **Search 1:**  TS=((cigarette OR conventional cigarette OR ends OR electronic nicotine delivery system OR electronic cigarette OR e-cigarette OR heat-not-burn product OR heat-not burn cigarette OR tobacco heating product OR heated tobacco products OR snus OR Swedish snuff OR oral nicotine pouches OR nicotine pouches) AND (health OR health effect OR health impact))  Filters: Document types: Articles; Languages: English; Open Access: All OA | 747,652 |
|  | **Search 2:**  TS= ((cigarette OR electronic cigarette OR e-cigarette OR heat-not-burn OR heated tobacco product OR snus OR oral nicotine pouches) AND (health effect OR health impact))  Filters: Document types: Articles; Languages: English; Open Access: All OA; Search within all fields: Human studies | 6,729 |
| **The Cochrane Database of Systematic Reviews (CDSR)** | **Search 1:**  (cigarette OR conventional cigarette OR ends OR electronic nicotine delivery system OR e-cigarette OR electronic cigarette OR heated tobacco product OR heat-not-burn OR snus OR oral nicotine pouches OR nicotine pouches) ti,ab,kw AND (health effects OR health impact) ti,ab,kw | 247,134 |
|  | **Search 2:**  (cigarette OR electronic cigarette OR e-cigarette OR heated tobacco product OR heat-not-burn OR snus OR oral nicotine pouches OR nicotine pouches)ti,ab,kw AND (health effects OR health impact)ti,ab,kw | 10,861 |
