# Supplementary material for: Comparison of the health-related outcomes for traditional cigarettes, e-cigarettes, heat-not-burn cigarettes and snus: a systematic review and meta-analysis
Source: BMC Public Health. 2026 Mar 26;26:1458. doi: 10.1186/s12889-026-27067-z (PMC13141496; doi:10.1186/s12889-026-27067-z)
Supplement: Supplementary file 2 — Supplementary Material 2. [file 12889_2026_27067_MOESM2_ESM.docx]

**Table S2. Summary table detailing the number of studies and participants per product and outcome.**

| Smoking products | Outcomes | No. of studies | Total participants (n) |
| --- | --- | --- | --- |
| Acute smoking | | | |
| TC | SBP | 1 | 100 |
| TC+EC |  | 1 | 90 |
| EC |  | 4 | 194 |
| HTP |  | 2 | 146 |
| snus |  | 1 | 58 |
| TC | DBP | 1 | 100 |
| TC+EC |  | 1 | 90 |
| EC |  | 4 | 194 |
| HTP |  | 2 | 146 |
| snus |  | 1 | 58 |
| TC | HRT | 1 | 100 |
| TC+EC |  | 1 | 90 |
| EC |  | 4 | 194 |
| HTP |  | 2 | 146 |
| snus |  | 1 | 58 |
| TC | FEV_1_ | 1 | 98 |
| TC+EC |  | 1 | 90 |
| EC |  | 2 | 120 |
| HTP |  | 1 | 98 |
| TC | FVC | 1 | 98 |
| TC+EC |  | 1 | 90 |
| EC |  | 1 | 90 |
| HTP |  | 1 | 98 |
| Chronic smoking | | | |
| TC | total cholesterol levels | 4 | 851 |
| TC+EC |  | 1 | 123 |
| EC |  | 1 | 127 |
| TC | triglyceride levels | 4 | 851 |
| TC+EC |  | 1 | 123 |
| EC |  | 1 | 127 |
| TC | glucose levels | 2 | 433 |
| TC+EC |  | 1 | 123 |
| EC |  | 1 | 127 |
| TC | SBP | 3 | 172 |
| EC |  | 1 | 46 |
| TC | DBP | 3 | 172 |
| EC |  | 1 | 46 |
| TC | HRT | 2 | 124 |
| EC |  | 1 | 46 |
| TC | FEV_1_ | 8 | 3496 |
| EC |  | 2 | 76 |
| HTP |  | 1 | 1555 |
| TC+HTP |  | 1 | 1551 |
| TC | FVC | 6 | 1639 |
| EC |  | 2 | 76 |
| Risk of cancer development | | | |
| cigarettes and/or other tobacco products | risk of cancer | 11 | 63668 |

*Abbreviations: TC – traditional cigarette; EC – electronic cigarette; HTP – heated tobacco product; SBP – systolic blood pressure; DBP – diastolic blood pressure; HRT – heart rate; FEV_1_ – forced expiratory volume in one second; FVC – forced vital capacity.*
