# Supplementary figures and images for: Comparison of the health-related outcomes for traditional cigarettes, e-cigarettes, heat-not-burn cigarettes and snus: a systematic review and meta-analysis
Source: BMC Public Health. 2026 Mar 26;26:1458. doi: 10.1186/s12889-026-27067-z (PMC13141496; doi:10.1186/s12889-026-27067-z)

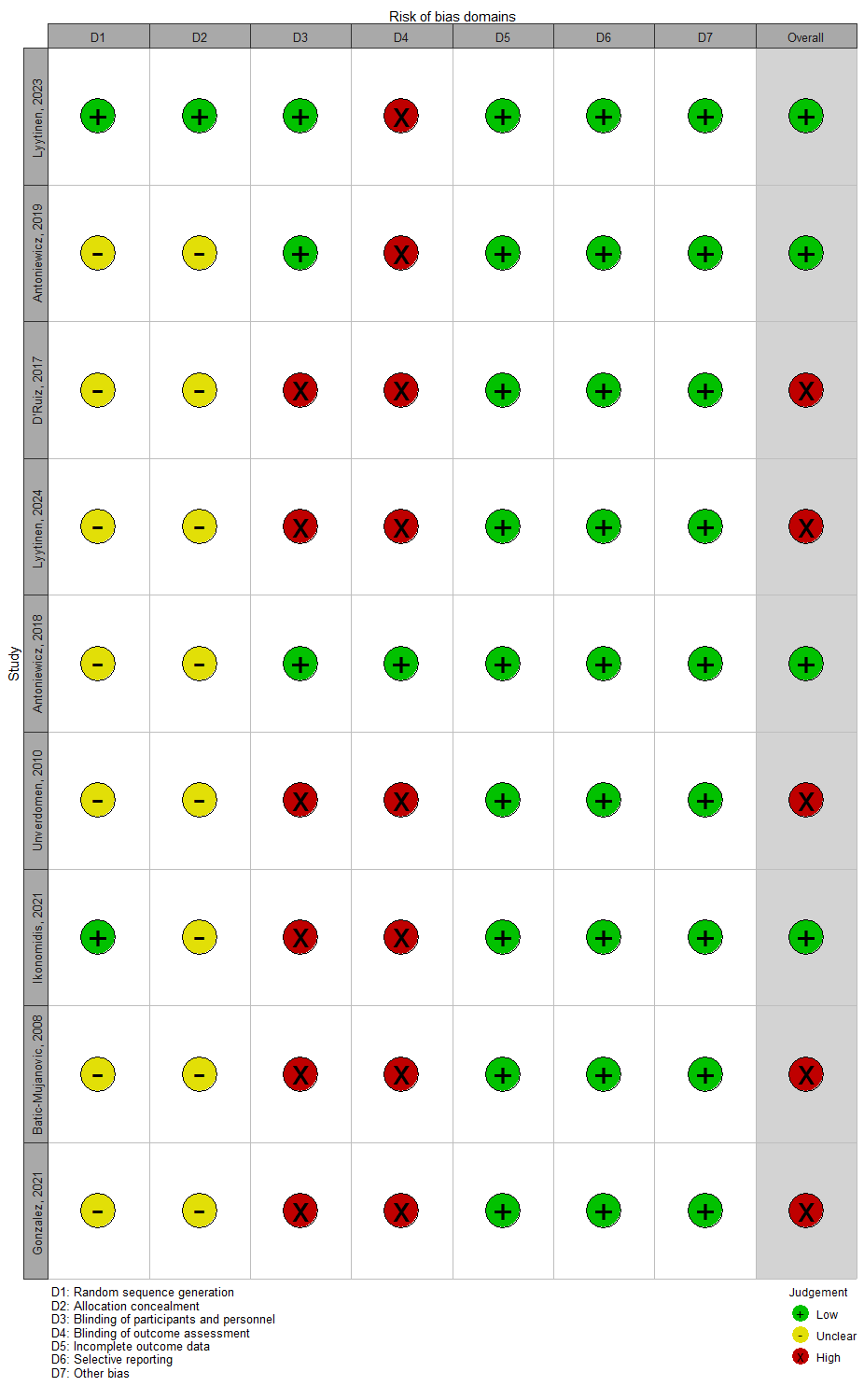
**Figure S1. Risk of bias of included RCTs.**

Supplement: Supplementary file 3 — Supplementary Material 3. [file 12889_2026_27067_MOESM3_ESM.docx]

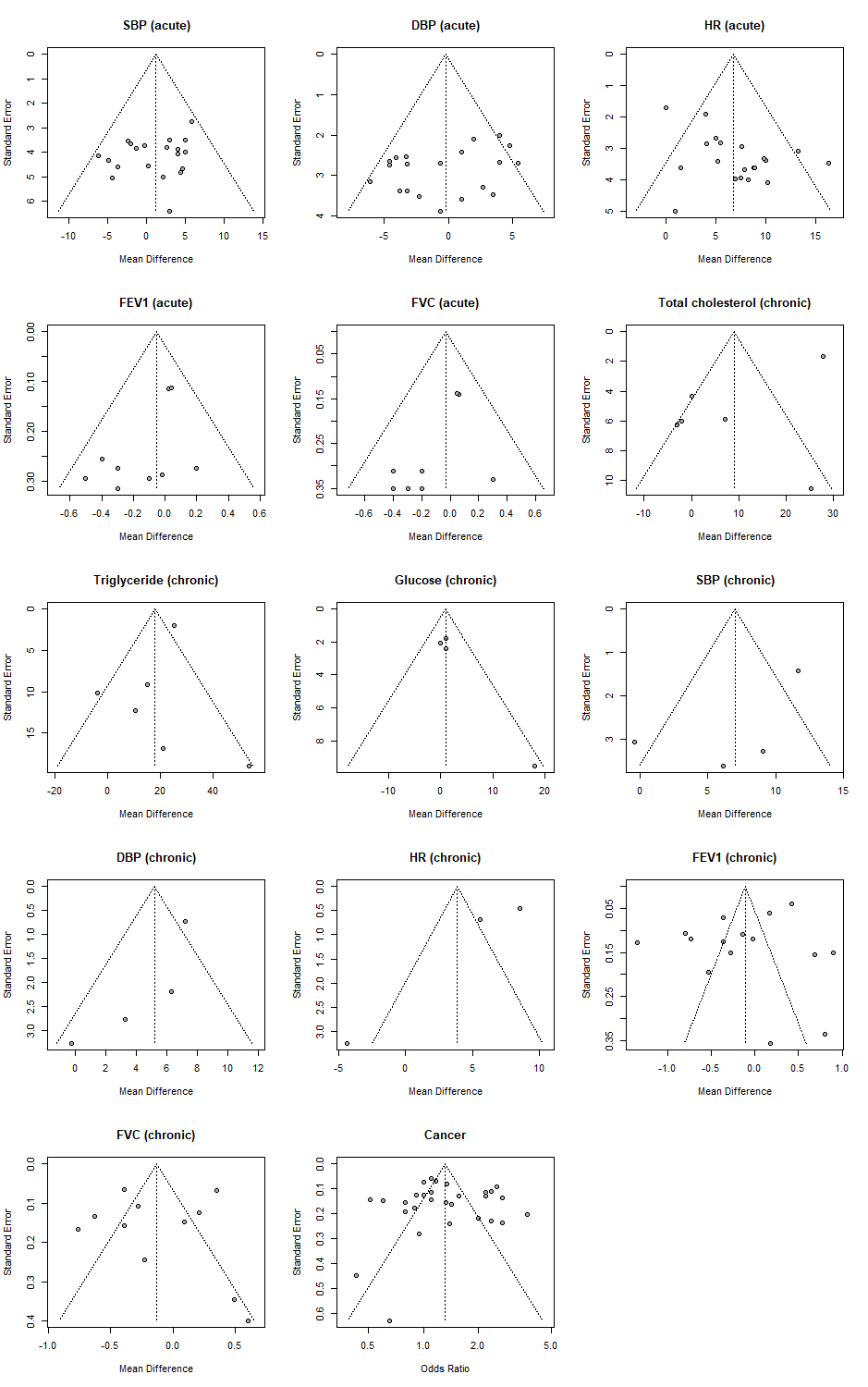


**Figure S2. Funnel plots for the association between smoking and outcomes.**

Supplement: Supplementary file 6 — Supplementary Material 6. [file 12889_2026_27067_MOESM6_ESM.docx]
