# Supplementary material for: Comparison of the health-related outcomes for traditional cigarettes, e-cigarettes, heat-not-burn cigarettes and snus: a systematic review and meta-analysis
Source: BMC Public Health. 2026 Mar 26;26:1458. doi: 10.1186/s12889-026-27067-z (PMC13141496; doi:10.1186/s12889-026-27067-z)
Supplement: Supplementary file 4 — Supplementary Material 4. [file 12889_2026_27067_MOESM4_ESM.docx]

**Table S3. The quality assessment of the included case-control studies using the Newcastle-Ottawa Scale.**

|  | **Selection** | | | | **Comparability** | **Exposure** | | | **Total** |
| --- | --- | --- | --- | --- | --- | --- | --- | --- | --- |
|  | Is the case definition adequate? | Representativeness of the cases | Selection of controls | Definition of controls | Comparability of cases and controls on the basis of the design or analysis | Ascertainment of exposure | Same method of ascertainment for cases and controls | Non-response rate |  |
| Kessides *et al.,* 2010 | ***** | ***** | ***** | ***** | ****** |  | ***** | ***** | **8** |
| Jiang *et al.*, 2012 | ***** | ***** | ***** |  | ****** |  | ***** |  | **6** |
| Modugno *et al.,* 2002 | ***** | ***** | ***** |  | ****** |  | ***** | ***** | **7** |
| Connor *et al.*, 2016 | ***** | ***** | ***** |  | ****** |  | ***** |  | **6** |
| Morton *et al.*, 2003 | ***** | ***** | ***** |  | ****** |  | ***** | ***** | **7** |
| Hjalgrim *et al.*, 2007 | ***** | ***** | ***** | ***** | ****** |  | ***** | ***** | **8** |
| Chang *et al.*, 2017 | ***** | ***** | ***** |  | ****** |  | ***** | ***** | **7** |
| Yang *et al.*, 2010 | ***** | ***** | ***** | ***** | ****** |  | ***** | ***** | **8** |
| Walter *et al.,* 1979 | ***** | ***** | ***** | ***** | ****** |  | ***** |  | **7** |
| Habib *et al.,* 2011 | ***** | ***** | ***** | ***** | ****** |  | ***** |  | **7** |
| Barter *et al.,* 1985 | ***** | ***** | ***** | ***** | ****** |  | ***** |  | **7** |
| Byerley *et al.,* 1992 | ***** | ***** | ***** |  | ****** |  | ***** |  | **6** |
| Tantisuwat and Thaveeratitham, 2014 | ***** | ***** | ***** | ***** | ****** |  | ***** |  | **7** |
