# Supplementary material for: Comparison of the health-related outcomes for traditional cigarettes, e-cigarettes, heat-not-burn cigarettes and snus: a systematic review and meta-analysis
Source: BMC Public Health. 2026 Mar 26;26:1458. doi: 10.1186/s12889-026-27067-z (PMC13141496; doi:10.1186/s12889-026-27067-z)
Supplement: Supplementary file 5 — Supplementary Material 5. [file 12889_2026_27067_MOESM5_ESM.docx]

**Table S4. The quality assessment of the included cross-sectional studies using the AXIS tool.**

| **No.** | **Questions to consider** | **Sobczak *et al.,* 2014** | **Boakye *et al.,* 2023** | **Wu *et al.,* 2020** | **Ellis *et al.,* 2024** | **Meo *et al.,* 2019** | **Milaat and el-Ganai, 1998** | **Jawed *et al.,* 2012** | **Harada *et al.,* 2021** | **Kizhakke Puliyakote *et al.,* 2021** | **Chandala and Reddy, 2014** | **Majid *et al.,* 2021** |
| --- | --- | --- | --- | --- | --- | --- | --- | --- | --- | --- | --- | --- |
| 1 | Were the aims/objectives of the study clear? | **+** | **+** | **+** | **+** | **+** | **+** | **+** | **+** | **+** | **+** | **+** |
| 2 | Was the study design appropriate for the stated aim(s)? | **+** | **+** | **+** | **+** | **+** | **+** | **+** | **+** | **+** | **+** | **+** |
| 3 | Was the sample size justified? | **?** | **?** | **?** | **?** | **-** | **-** | **+** | **-** | **-** | **-** | **-** |
| 4 | Was the target/reference population clearly defined?  (Is it clear who the research was about?) | **+** | **+** | **+** | **+** | **+** | **+** | **+** | **+** | **+** | **+** | **+** |
| 5 | Was the sample frame taken from an appropriate population base so that it closely represented the target/reference population under investigation? | **-** | **+** | **+** | **+** | **+** | **+** | **+** | **+** | **+** | **+** | **+** |
| 6 | Was the selection process likely to select subjects/participants that were representative of the target/reference population under investigation? | **+** | **+** | **+** | **+** | **+** | **+** | **+** | **+** | **+** | **+** | **+** |
| 7 | Were measures undertaken to address and categorize non-responders? | **?** | **?** | **?** | **?** | **-** | **-** | **-** | **-** | **-** | **-** | **-** |
| 8 | Were the risk factor and outcome variables measured appropriate to the aims of the study? | **+** | **+** | **+** | **+** | **+** | **+** | **+** | **+** | **+** | **+** | **+** |
| 9 | Were the risk factor and outcome variables measured correctly using instruments/measurements that had been trialed, piloted or published previously? | **+** | **+** | **+** | **+** | **+** | **+** | **+** | **+** | **+** | **+** | **+** |
| 10 | Is it clear what was used to determined statistical significance and/or precision estimates? (e.g., p values, Cis) | **+** | **+** | **+** | **+** | **+** | **+** | **+** | **+** | **+** | **+** | **+** |
| 11 | Were the methods (including statistical methods) sufficiently described to enable them to be repeated? | **+** | **+** | **+** | **+** | **+** | **+** | **+** | **+** | **+** | **+** | **+** |
| 12 | Were the basic data adequately described? | **+** | **+** | **+** | **+** | **+** | **+** | **+** | **+** | **+** | **+** | **+** |
| 13 | Does the response rate raise concerns about non-response bias? | **-** | **-** | **-** | **-** | **?** | **?** | **?** | **?** | **?** | **?** | **?** |
| 14 | If appropriate, was information about non-responders described? | **+** | **+** | **-** | **+** | **-** | **-** | **-** | **-** | **-** | **-** | **-** |
| 15 | Were the results internally consistent? | **+** | **+** | **+** | **+** | **+** | **+** | **+** | **+** | **+** | **+** | **+** |
| 16 | Were the results for the analyses described in the methods, presented? | **+** | **+** | **+** | **+** | **+** | **+** | **+** | **+** | **+** | **+** | **+** |
| 17 | Were the authors’ discussions and conclusions justified by the results? | **+** | **+** | **+** | **+** | **+** | **+** | **+** | **+** | **+** | **+** | **+** |
| 18 | Were the limitations of the study discussed? | **+** | **+** | **+** | **+** | **+** | **+** | **+** | **+** | **+** | **-** | **+** |
| 19 | Were there any funding sources or conflicts of interest that may affect the authors’ interpretation of the results? | **?** | **?** | **-** | **-** | **-** | **?** | **-** | **-** | **-** | **?** | **?** |
| 20 | Was ethical approval or consent of participants attained? | **+** | **+** | **+** | **+** | **+** | **+** | **-** | **+** | **+** | **-** | **+** |
